# Sustainable Thermoplastic Elastomers from Commercial Cyclic Esters: One-Pot Synthesis of Poly(l‑lactide)‑*b*‑(δ-hexalactone-*stat*-ε-caprolactone)‑*b*‑(l‑lactide) and Its Chemical Recycling to the Monomers

**DOI:** 10.1021/acspolymersau.5c00172

**Published:** 2026-01-08

**Authors:** Giuseppe Gravina, Joseph N. A. Tagoe, Rocco Di Girolamo, Maria Gentile, Luciano Di Maio, Claudio Pellecchia

**Affiliations:** † Dipartimento di Chimica e Biologia “A. Zambelli”, 19028 Università degli Studi di Salerno, via Giovanni Paolo II 132, Fisciano, SA 84084, Italy; ‡ Dipartimento di Ingegneria Industriale, 534774 Università degli Studi di Salerno, via Giovanni Paolo II 132, Fisciano, SA 84084, Italy; § Dipartimento di Scienze Chimiche, 9307 Università degli Studi di Napoli Federico II, Via Cintia 21, Napoli 80126, Italy; ∥ Dipartimento di Ingegneria Industriale, Università di Padova, via Gradenigo, 6/a, Padova, PD 35131, Italy

**Keywords:** ring opening polymerization, triblock copolymers, thermoplastic elastomers, sustainable polymers, chemical recycling to monomer

## Abstract

We report the *one-pot* synthesis of ABA triblock copolymers composed of poly­(l-lactide) (PLA) as hard segments and δ-hexalactone/ε-caprolactone (HL/CL) copolymers as soft midblock. Use of an environmentally benign and highly active and living Fe­(II)-based catalyst enabled precise molecular design and control over copolymer architecture, providing low-*T*_g_, amorphous statistical HL/CL dihydroxy-terminated copolymers, which could be chain extended with l-lactide in the same reactor, resulting in poly­(l-lactide)-*b*-(δ-hexalactone-*stat*-ε-caprolactone)-*b*-(l-lactide) triblock copolymers. The *one-pot* synthetic route enabled control of block composition and length, resulting in material property profiles ranging from silicone-like soft elastomers to SBS-like rubbers, and to toughened PLA. Finally, efficient chemical recycling to the pristine monomers with >90% recovery was achieved by catalytic thermal depolymerization under mild conditions.

## Graphical abstract

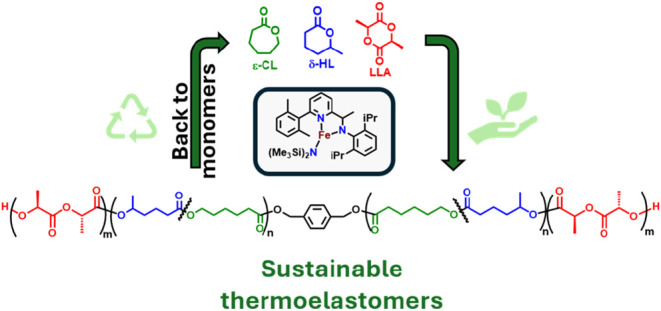

## Introduction

The transition to a more sustainable polymer production is particularly critical for rubbers, which are mostly chemically cross-linked polymeric materials, infusible and insoluble, therefore intrinsically nonrecyclable. For some applications, an alternative to vulcanized rubbers is represented by thermoplastic elastomers (or thermoelastomers, TPE’s), which combine the fundamental properties of rubbers with those of thermoplastic polymers and are reprocessable and recyclable.^1^ One of the most important commercial TPE’s is SBS rubber, a styrene–butadiene-styrene triblock copolymer, consisting of two “hard”, high-*T*_g_, polystyrene side blocks and a “soft” amorphous, low-*T*_g_ polybutadiene central block.^2^ The elastic behavior of SBS rubber is due to the immiscibility of the polystyrene and polybutadiene phases, linked together by physical cross-links consisting of the polystyrene-rich phases that remain rigid up to *T* ≈ 100 °C while the polybutadiene-rich phases remain flexible up to *T* ≈ −80 °C. Unlike chemical cross-links in vulcanized rubbers, physical cross-links in SBS are thermally cleavable because above its *T*_g_ polystyrene becomes fluid and the material can be processed and reprocessed as a thermoplastic polymer.

However, SBS is obtained from raw materials of fossil origin and is not biodegradable. For a greater sustainability of rubbers toward the transition to a circular economy, it is desirable to produce thermoelastomers based on renewable raw materials (e.g., biomass from agro-industrial waste), biodegradable and recyclable.^3^ Extensive research has focused on ABA triblock copolymers based on aliphatic polyesters, which can be obtained from biobased monomers, usually have inherent hydrolytic degradability, and can be chemically recycled to the monomers.^4^ Such ABA copolyesters can be synthesized through ring-opening polymerization (ROP) of cyclic esters, which allows to efficiently control the molecular architecture, composition and relative length of the blocks to modulate the thermal, mechanical and degradation properties of the produced materials. Poly­(lactic acid) or poly­(lactide) (PLA) has been widely explored as a sustainable replacement for polystyrene (PS), and its use has been extended to serve as the *hard* A segments in ABA triblock thermoelastomers.^5^ Although both atactic and isotactic PLA A-end blocks have been used, better performance was observed for isotactic, semicrystalline PLA-based TPEs, owing to the rather low *T*_g_ of atactic PLA.^6^ The selection of the soft aliphatic polyester midblock incorporated into PLA-containing TPEs is more critical: particularly relevant for this issue are the contributions by Hillmyer and co-workers, who tested a variety of amorphous aliphatic polyesters, including poly­(6-methyl-ε-caprolactone),^7^ poly­(β-methyl-δ-valerolactone),^8^ poly­(menthide),^6^ resulting in some cases in the production of TPE’s with excellent performance. However, most of the used lactone monomers are not commercially available and must be synthesized *ad hoc* either from fossil sources or prepared by biofermentation with a genetically modified bacterium.^6–8^

On the other hand, a convenient candidate, for cost and availability, could be poly­(ε-caprolactone) (PCL), a commercial polymer with a *T*_g_ of ≈ −60 °C, which however is semicrystalline, with a *T*_m_ of ≈60 °C, and therefore it is rigid at room temperature, resulting in the production of ABA triblock copolymers (A= PLA) with limited elastomeric performance.^9^ To reduce or suppress the crystallinity of the PCL based midblock, copolymers of ε-caprolactone with different cyclic esters were also tested. E.g., a poly­(ε-caprolactone-*co*-δ-valerolactone) midblock was used to produce ABA triblock TPE’s by chain extension with L-LA or D-LA.^10^ However, owing to the cocrystallization of ε-CL and δ-VL units in a single crystalline phase, the copolymer retains some crystallinity, which, although could be suppressed by suitable thermal treatments, can still develop during the material lifetime, possibly affecting the mechanical properties. Also, dihydroxy-terminated pseudorandom copolymers of ε-CL with *rac*-lactide were successfully used as macroinitiators to produce poly­(L-LA)-*b-*poly­(ε-CL-*co*-D,L-LA)*-b-*poly­(L-LA) ABA triblock copolymers having good elastomeric properties, although the different reactivity ratios of the two monomers, resulting in a relatively high content of *rac*-lactide needed to suppress PCL crystallinity, lead to a significant increase of the *T*_g_ of the soft midblock (*T*_g_ > −30 °C).^11^ Dihydroxyl-end-capped telechelic ε-caprolactone-*co*-ε-decalactone copolymers, having a gradient structure owing to the different monomer reactivity ratios, were also used to prepare ABA triblock thermoelastomers with PLLA hard end blocks.^12^

δ-Substituted δ-lactones are naturally occurring cyclic esters with a six-membered ring and an alkyl substituent in the δ position: δ-hexalactone (or δ-caprolactone), δ-heptalactone, δ-octalactone, δ-nonalactone, δ-decalactone, δ-undecalactone, δ-dodecalactone, and δ-tetradecalactone are all commercially available on a significant scale because they are used as flavors or fragrances in the food or cosmetic industries and can be extracted from vegetables or obtained from biomass. These lactones were less investigated as potential monomers for the production of sustainable polyesters, because they were considered difficult to efficiently undergo ROP due to the low steric strain of the six-membered ring and the bulkiness of the δ-substituent, resulting in low ceiling temperatures (*T*_c_) of their polymers.^13^ However, they have recently attracted some attention,^14^ following the increasing interest for low *T*_c_ polymers in view of the possibility of achieving easy chemical recycling to the monomers.^15^ Actually, efficient ROP of δ-hexalactone (δ-HL) using strong base/urea organocatalysts and the preparation of PLA-*b*-PδHL-*b*-PLA triblock copolymers with thermoplastic elastomer properties have been recently reported.^16^

In the context of our search for new efficient ROP catalysts based on nontoxic and earth-abundant metals,^17–21^ we have recently synthesized 3-coordinated Fe­(II) complexes bearing pyridylamido ligands with bulky substituents at both the pyridino and amido moieties and we have used them as highly active and living catalysts for the ROP of l-lactide, ε-caprolactone,^22^ and various δ-substituted δ-lactones.^23^ In this work, we report the copolymerization of δ-hexalactone with ε-caprolactone, affording amorphous, low *T*_g_ statistical copolymers, which were chain extended with L-LA using a *one-pot* procedure affording triblock polymers of different compositions. Depending on the latter, the polymers can show mechanical properties comparable to those of commercial SBS rubbers (e.g., tensile strength up to 9 MPa, strain at break >800%, elastic recovery >90%, Young’s modulus up to 26 MPa), or to those of “soft” elastomers such as polydimethylsiloxane (e.g., tensile strength <1 MPa, strain at break >150%, Young’s modulus ≈ 4 MPa), or to those of high-impact polystyrene (e.g., Young’s modulus ≈ 0.9 GPa, yield strength >25 MPa, tensile strength at break up to 24 MPa, strain at break ≈300%). A key aspect of this study is the combination of an iron-based living catalytic system with a *one-pot* sequential strategy, which enables the straightforward construction of ABA triblock architectures without intermediate purification. This approach represents a significant methodological advance, as it couples the use of an earth-abundant, low-toxicity metal catalyst with an efficient and operationally simple route to produce well-defined block copolymers. Moreover, δ-substituted lactones—naturally occurring and available on an industrial scale—are shown here to generate amorphous, low-*T*_g_ copolymers being ideally suitable midblocks for thermoplastic elastomers, overcoming limitations associated with more conventional lactones. An additional strength of the system is its intrinsic chemical recyclability: the triblock copolymers can be depolymerized under mild catalytic conditions, enabling near-quantitative monomer recovery and demonstrating the feasibility of a circular material design.

## Experimental Section

### Experimental Details

All procedures involving compounds sensitive to air and moisture were carried out under a nitrogen atmosphere, either in a Braun Labmaster glovebox or by employing standard Schlenk techniques. Glassware was predried in an oven at 120 °C overnight and subsequently subjected to alternating vacuum-nitrogen cycles. Solvents were purified as follows: toluene was refluxed over metallic sodium and distilled under nitrogen prior to use. Deuterated solvents were purchased from Sigma-Aldrich and stored in the glovebox over 4 Å molecular sieves until required. δ-Hexalactone, and ε -caprolactone (Sigma-Aldrich) were stirred with CaH_2_ for 24 h at room temperature and then distilled under reduced pressure before use. The lactide used was purified through recrystallization from anhydrous toluene. The pyridylamino ligand^24^ and the corresponding pyridylamido Fe­(II) complex^22^ (**Fe-1**, see Scheme 1) were synthesized as previously reported. All other reagents were commercially sourced and used without further purification unless otherwise specified.

**Scheme 1:**
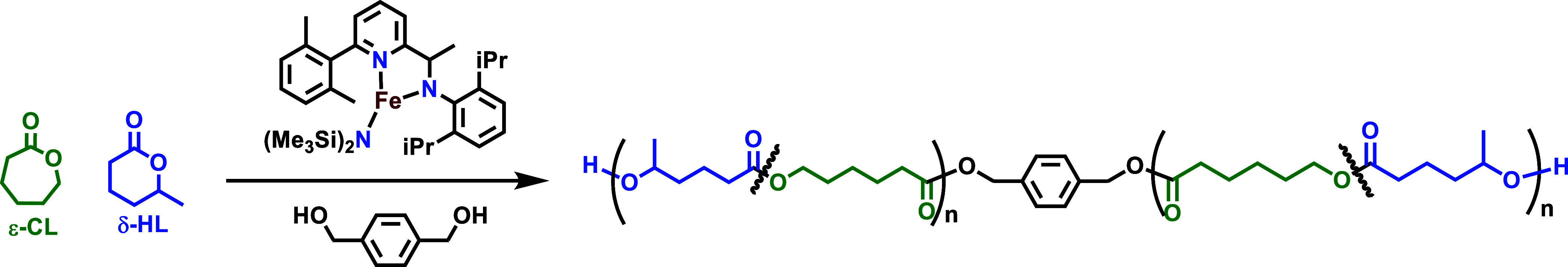
Copolymerization of δ-Hexalactone with ε-Caprolactone

NMR spectra were collected on either a Bruker Advance 400 or a 600 MHz Ascend 3 HD spectrometer. Chemical shifts (δ) are reported in parts per million (ppm) and coupling constants (*J*) in hertz (Hz). ^1^H NMR spectra were referenced against the residual solvent peaks at δ = 7.26 for CDCl_3_, δ = 5.32 for CD_2_Cl_2_, and δ = 7.16 for C_6_D_6_.

The number-average (*M*_n_) and weight-average (*M*_w_) molecular weights, along with the molecular weight distribution (*M*_w_/*M*_n_), of polymer samples were determined by gel permeation chromatography (GPC) using an Agilent 1260 Infinity Series instrument (ResiPore 3 μm, 300 mm × 7.5 mm column, 1.0 mL min^–1^ flow rate) equipped with a refractive index (RI, PLGPC 220) detector. All analyses were performed in THF as the eluent at 35 °C with a flow rate of 1.0 mL/min. Monodisperse polystyrene standards were employed for calibration.

Differential scanning calorimetry (DSC) was carried out on a TA Q20 instrument, calibrated for both temperature and enthalpy using a high-purity indium standard (156.60 °C, 28.45 J g^–1^). Thermogravimetric analysis (TGA) was performed on a Q500 analyzer from 25 to 800 °C, at a heating rate of 20 °C/min, under nitrogen flow.

Wide-angle X-ray diffraction (WAXD) pattern collected on the δ-hexalactone/ε-caprolactone (HL/CL) copolymer was obtained using an automatic Bruker D2 phaser diffractometer, in reflection geometry, at 35 kV and 40 mA, using nickel-filtered Cu Kα radiation (1.5418 Å).

Simultaneous Small Angle X-ray Scattering (SAXS) and Wide Angle X-ray diffraction (WAXD) synchrotron measurements have been performed on selected block copolymers at Dutch-Belgian beamline BM26-Dubble of ESRF (Grenoble, France). The wavelength of incident X-rays was λ = 0.950 nm. The covered range for the scattering vector *q* was 0.1–4 nm^–1^ for SAXS and 5–50 nm^–1^ for WAXD. The temperature was controlled using a Linkam DSC cell. SAXS and WAXD data were collected in the melt state at 200 °C and during cooling from the melt to 25 °C (cooling rate: 10 °C/min), acquiring one frame every 6 s (≈1 frame per °C). SAXS and WAXD experiments were also performed on samples crystallized from solution. One-dimensional profiles were extracted from the two-dimensional images using Bubble software.

### Synthesis of Poly­(δ-hexalactone-*stat*-ε-caprolactone)

In a typical procedure, in a 20 mL glass reactor, 4.2 mg (30 μmol) of 1,4-benzenedimethanol were charged, followed by 1.9 g δ-hexalactone (δ-HL, 16.6 mmol), 0.82 g ε-caprolactone (ε-CL, 7.20 mmol), and 18 mg of **Fe-1** in 0.5 mL toluene. The mixture was stirred at room temperature, and monomer conversions were monitored by ^1^H NMR analysis of aliquots collected at appropriate time intervals. After 6 h, the conversions reached 95% for ε-CL and 85% for δ-HL. At this point, the reaction mixture was poured into 400 mL of cold, HCl-acidified methanol. The resulting solid was isolated by filtration and dried under vacuum at 50 °C for 18 h, affording 1.8 g of product.

### Synthesis of Poly­(l-lactide)-*b*-(δ-hexalactone-*stat*-ε-caprolactone)-*b*-(l-lactide) Samples

Detailed experimental procedures for the triblock copolymers reported in Table 2 are outlined below. Polymer 1 was synthesized by repeating the procedure described above for the random HL/CL copolymer. Subsequently, l-lactide (0.47 g, 3.25 mmol) dissolved in 20 mL of anhydrous CH_2_Cl_2_ was added to the reaction mixture. The mixture was stirred at room temperature for 14 days, then poured into 400 mL of HCl-acidified methanol. The resulting precipitate was collected by filtration and dried under vacuum at 50 °C for 18 h, yielding 2.8 g of polymer. Polymer 2 was prepared in the same way, except that only 0.235 g (1.62 mmol) of l-lactide was added in the second step. For Polymer 3, the first step followed the same procedure, while the second step was carried out at 80 °C, with magnetic stirring for 12 h. Polymer 4 was synthesized as follows: in a 20 mL glass reactor, 1.4 mg (10 μmol) of 1,4-benzenedimethanol were combined with 0.17 g of δ-hexalactone (15 mmol), 0.17 g of ε-caprolactone (15 mmol), and 6 mg of **Fe-1** dissolved in 0.2 mL of toluene. After stirring at room temperature for 2 h, an aliquot was analyzed by ^1^H NMR, confirming nearly complete conversion of both monomers. l-lactide (0.86 g, 6.0 mmol) and 10 mL of anhydrous CH_2_Cl_2_ were then added, and the mixture was stirred for 6 days before being worked up as described above (yield: 1.0 g).

All samples were analyzed by ^1^H NMR. The relative lengths of the hard and soft blocks were determined from the spectra by integrating the resonances of the PLA blocks and those of the P­(HL-*stat*-CL) block relative to the resonances of the benzenedimethanol initiator. The volume fraction of the hard blocks was calculated using the density of PLA and assuming that the density of P­(HL-*stat*-CL) is similar to that of PCL.

### Mechanical Properties of Poly­(l-lactide)-*b*-(δ-hexalactone-*stat*-ε-caprolactone)-*b*-(l-lactide) Samples

Tensile tests were performed on polymer films obtained by solvent casting: a known quantity of each polymer was dissolved in CH_2_Cl_2_ to form the casting solutions with a concentration of 2 %wt/wt. The solutions were then cast onto a Petry dish with smooth, nonstick surface. The resulting films were allowed to dry overnight under a fume hood to ensure complete and proper solvent evaporation yielding films with a thickness of 0.08–0.10 mm (Figure S17).

Conditioning: prior to testing, all specimens were conditioned for a minimum of 40 h at a standard atmosphere of 23 °C ± 2 °C and 50% RH, as required by the ASTM D882 standard specifications. Sample dimensions: samples were carefully cut into rectangular strips with a uniform width of 10 mm and a length of 80 mm. The thickness of each specimen was confirmed to be uniform by measuring multiple points using a micrometer screw gauge. Testing: a minimum of 5 specimens were tested for each film composition.

Tensile tests were performed using a CMT 4000 Series tensile tester (SANS, China) equipped with a 1 KN load cell. To ensure accurate data integrity and mitigate artifacts like sample slippage and grip-induced damage, the tensile tester was fitted with high-precision, pneumatically actuated antislippage clamps. This system delivers a secure, uniform grip across the entire sample width, effectively preventing relative movement during elongation. The pneumatic controller allows for precise, repeatable adjustment of the clamping pressure. The clamping force was optimized empirically to generate sufficient friction for a firm, nonslip hold (adequate to handle the material’s ultimate tensile strength) while remaining below the threshold that would induce stress concentration or premature failure at the grip interface. This methodology ensures film failure occurs reliably within the gauge section.

It is important to acknowledge that the tensile properties (e.g., Young’s Modulus, ultimate tensile strength) of polymeric materials are typically strain-rate dependent. Although a detailed strain rate sensitivity analysis is relevant, it fell outside the scope of this study, which centered on material synthesis and baseline characterization. Consequently, the mechanical testing was executed under standardized, reproducible conditions to establish a baseline for comparison with published data. For these highly deformable materials, the chosen methodology adheres to ASTM D882 and the standard practice of using two distinct strain rates: a slower rate of 30 mm/min crosshead speed for accurate measurement of small-deformation properties (e.g., elastic modulus), and a faster rate of 300 mm/min for the ultimate properties (stress and strain at break). These speeds were selected to yield consistent and standardized strain rates for the respective property measurements, considering the film geometry.

### Depolymerization of Poly­(l-lactide)-*b*-(δ-hexalactone-*stat*-ε-caprolactone)-*b*-(l-lactide)

In a representative experiment 3 mg of polymer 2 of Table 2 were placed into a thermogravimetric analysis (TGA) crucible, and 2 mol % (relative to ester linkages) of catalyst (either Sn­(Oct)_2_ or Zn­(Oct)­2) was added as a 0.02 M THF solution. After completing solvent evaporation, the crucible was loaded into the TGA instrument to monitor the depolymerization process. The TGA experiments were repeated under the same conditions described above, but adding glycerol-ethoxylate (GEO) as solution containing 10 equiv of hydroxyl groups relative to the catalyst. The kinetic constants, determined from the slope of the linear fits to the plots of polymer mass loss (%) versus time, are summarized in Table S1. The TGA program used for the experiments consisted of temperature ramp from room temperature to 180 °C, an isothermal hold for 100 min, a heating ramp of 30 °C min^–1^ up to 700 °C, followed by equilibration at 30 °C.

For the bulk depolymerization experiments, 100 mg of poly­(l-lactide)-*b*-(δ-hexalactone-*stat*-ε-caprolactone)-*b*-(l-lactide) (polymer 2 of Table 2), 2 mol % of Sn­(Oct)_2_ (with respect to ester linkages), and GEO were introduced into a sublimator equipped with magnetic stirring, ensuring a [OH]/[catalyst] molar ratio of 10:1. The reactions were carried out at 180 °C under vacuum (<1 Torr). After 2 h, the reactor was cooled to room temperature, and the entire reaction mixture was dissolved in CHCl_3_. The solvent was then removed under reduced pressure, and the resulting solid residue was analyzed by ^1^H NMR spectroscopy to determine its composition.

## Results and Discussion

### Copolymerization of δ-Hexalactone and ε-Caprolactone

The binary copolymerization of δ-hexalactone with ε-caprolactone was investigated under different conditions using the previously reported 3-coordinated Fe­(II) pyridylamido complex as the catalyst and 1 equiv of benzenedimethanol as the co-initiator (see Scheme 1).

We first screened the copolymerization of δ-hexalactone (δ-HL) with ε-caprolactone (ε-CL) using different feed ratios and reaction conditions, targeting the production of low-*T*_g_ amorphous statistical copolymers. The results of some representative runs are displayed in Table 1. Bulk copolymerization at 30 °C using a 70:30 ε-CL/δ-HL feed ratio (run 1, Table 1) resulted in complete conversion of ε-CL in 2.5 h, while 40% of δ-HL remained unreacted. The resulting copolymer contains 85% ε-CL and it is semicrystalline (*T*_m_ = 46 °C), owing to the presence of long ε-CL sequences. Using a 50:50 ε-CL/δ-HL feed ratio under the same conditions afforded a copolymer containing 55% of ε-CL, still showing some crystallinity (run 2, Table 1). Finally, use of a 30:70 ε-CL/δ-HL feed ratio resulted in a completely amorphous copolymer with a *T*_g_ = −48 °C.

**Table 1:** Statistical Copolymerization of δ-Hexalactone and ε-Caprolactone Promoted by **Fe-1**

run^a^	[HL]/[CL] (feed)	[HL]/[CL] (copolymer)	*T* (°C)	time (h)	conv (%)	*M*_n,Theo_ ^b^ (kDa)	*M*_n,GPC_ ^c^ (kDa)	*Đ* ^c^	*T*_g_ ^d^ (°C)	*T*_m_ ^d^ (°C)	Δ*H*_m_ (J/g)
1	30/70	15/85	30	2.5	60 (1)	29	121	2.1	/	46	24
					97 (2)						
2	50/50	45/55	30	2.5	80 (1)	31	43	2.0	–54	42	16
					100 (2)						
3	70/30	67/33	30	2.5	86 (1)	31	24	1.8	–48	/	/
					100 (2)						
4	60/40	50/50	80	0.1	76 (1)	28	22	1.6	–50	/	/
					93 (2)						
5^e^	60/40	38/62	80	1	41 (1)	22	29	1.6	–58	28	24
					100 (2)						

a Reactions were performed using 5 μmol of **Fe-1** catalyst, 1 equiv of benzenedimethanol and a total amount of 300 equiv of monomers.

b Theoretical molecular weight = 114­[monomer]^0^/[alcohol]^0^ × average conversion.

c Experimental *M*_n_ and *M*_w_/*M*_n_ (*Đ*) values were determined by GPC analysis in THF vs polystyrene standards.

d Determined by DSC in the first heating, heating rate = 10 °C min^–1^.

e Reaction performed in solution of toluene with [HL] + [CL] = 2 M.

A 50:50 amorphous statistical copolymer was obtained in much shorter time (6 min) performing the reaction at 80 °C using a 40:60 ε-CL/δ-HL feed ratio and solvent-free conditions. On the contrary, a similar run carried out in 2 M toluene solution afforded a copolymer with some residual crystallinity (*T*_m_ = 28 °C).

The copolymers were characterized by NMR analysis. As a representative example, the ^1^H NMR spectrum with full resonance assignments of the copolymer produced in run 3 is displayed in Figure 1. Analysis of the ^13^C NMR spectrum provides better insight into the copolymer microstructure: in particular, the carbonyl carbons are very sensitive to the variation of the chemical environment (see Figure S1), allowing quantification of the homo- and heterosequences.^25,26^ Analysis of the sequence distribution resulted in a randomness parameter *R* = 0.75,^27^ indicating a statistical copolymer structure with a slightly higher proportion of homosequences compared to the ideal random case (*R* = 1), due to the higher reactivity of CL compared to HL.^23^ This microstructure results in the formation of amorphous copolymers, as confirmed by thermal analysis (Table 1) and by the absence of crystalline reflections in the WAXD patterns (Figure S2).

**Figure 1:**
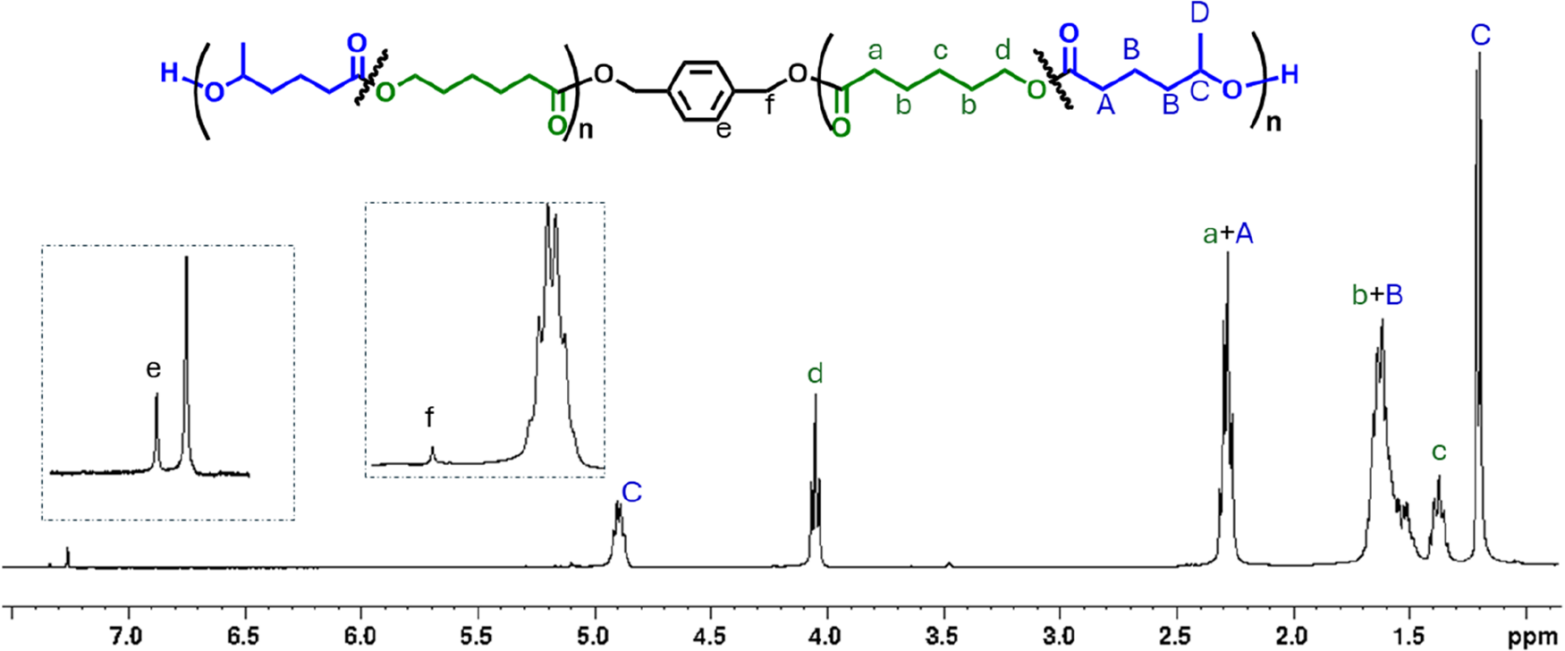
^1^H NMR (CDCl_3_, 600 MHz) spectrum of the HL/CL copolymer produced in run 3.

Thus, the composition of the copolymer produced in run 3 was selected as the low *T*_g_, amorphous midblock for the synthesis of triblock copolymers.

### One-Pot Synthesis of Triblock Poly­(l-lactide)-*b*-(δ-hexalactone-*stat*-ε-caprolactone)-*b*-(l-lactide)

ABA triblock copolymers were synthesized via a *one-pot* sequential reaction involving the preparation of a dihydroxyl-terminated δ-lactone-*stat*-ε-lactone copolymer followed by the addition of L-LA according to Scheme 2.

**Scheme 2:**
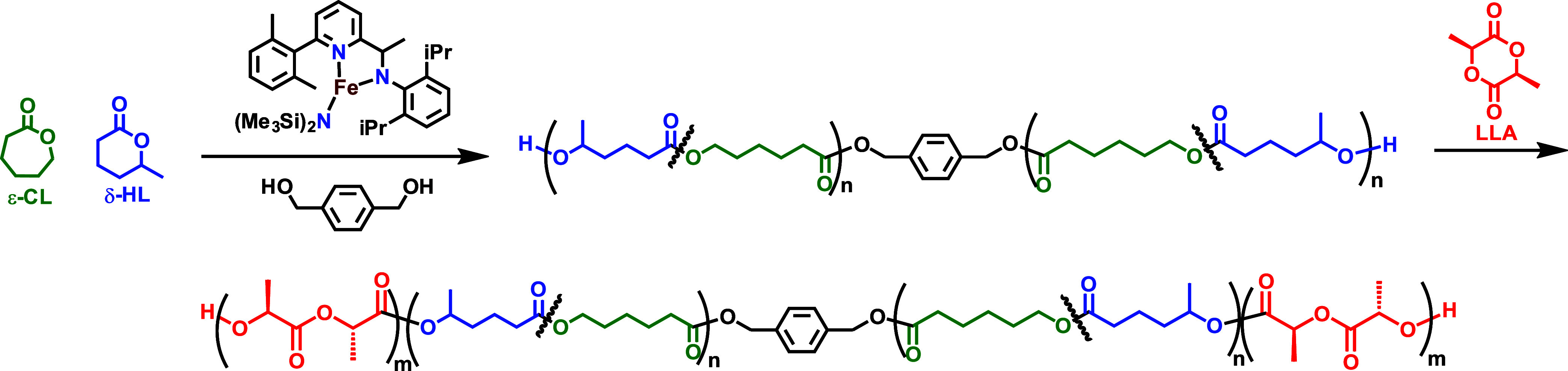
One-Pot Reaction Scheme for the Synthesis of ABA Triblock Polymers

Several ABA triblock polymers consisting of PLLA as semicrystalline hard A end blocks and statistical copolymers of δ-HL and ε-CL as amorphous, low *T*_g_ *soft* B block, were prepared following the above reaction scheme. In a typical experiment, a dihydroxyl-terminated poly­(δ-HL-*stat*-ε-CL) with a ≈60:40 δ-HL/ε-CL mol % composition was prepared in the first step, using **Fe-1** as the catalyst and 1 equiv of benzenedimethanol co-initiator in bulk at 30 °C. After collecting a small fraction of the reaction mixture for midblock characterization, a solution of L-LA was added to the same reactor and the mixture was stirred either at room temperature or at 80 °C, monitoring the conversion of lactide by withdrawing small samples and analyzing them by ^1^H NMR. Different runs were performed, varying the relative lengths of the *soft* and *hard* blocks. The resulting polymers were recovered as described in the Experimental Section and characterized by NMR, DSC, WAXD, SAXS, GPC, and tensile tests. The main characterization data are summarized in Table 2, discussed in the following paragraphs, and detailed in the Supporting Information (SI).

**Table 2:** Main Characterization Data of Poly­(l-lactide)-*b*-(δ-hexalactone-*stat*-ε-caprolactone)-*b*-(l-lactide) Samples

polymer sample	block length^a^	*f*_hard_ ^b^ %	*M*_n_ ^c^ (kDa)	*Đ* ^c^	*T*_g_ ^d^ (°C)	*T*_m_ ^d^ (°C)	Δ*H*_m_ ^d^ (J/g)	*E* ^e^ (Mpa)	ε^e^ (%)	σ^e^ (MPa)
1	60–270–60	35	85	1.2	–43	158	8	8.1 ± 0.9	874 ± 48	9.2 ± 1.9
2	30–270–30	17	70	1.3	–44	138	6	4.6 ± 2.3	167 ± 10	0.7 ± 0.2
3	80–330–80	34	103	1.3	–45	164	18	26 ± 1	697 ± 30	6.2 ± 0.1
4	120–90–120	72	77	1.2	–32	166	28	877 ± 37	296 ± 63	24.2 ± 1.6

a Esteemed from GPC and NMR analysis.

b Calculated volume fraction of PLLA hard phase.

c *M*_n_ and *M*_w_/*M*_n_ (*Đ*) values were determined by GPC analysis in THF vs polystyrene standards (see Figures S3–S5).

d Determined by DSC in the second heating, heating rate 10 °C min^–1^.

e Young’s modulus, strain at break and ultimate tensile strength measured by dynamometric uniaxial tensile tests (see Figure 6).

### Gel Permeation Chromatography (GPC) Analysis

The formation of the triblock polymers was evaluated by GPC by comparing the molecular weights of the central block and the corresponding final polymers. This analysis is crucial for demonstrating both the successful assembly of the triblock structures and the living nature of the catalyst—a point emphasized by the reviewers. Figures S3–S5 show the GPC overlays of the midblocks and their respective final polymers. The peak shapes remain essentially identical, the molecular weights increase consistently with the extent of LA conversion, and the dispersity is maintained—or even slightly reduced—from the midblock to the final polymer.

### Nuclear Magnetic Resonance (NMR) Analysis

The triblock polymers of Table 2 were extensively studied by NMR spectroscopy. The ^1^H NMR spectra allowed us to determine the molar monomer composition of the triblock copolymer and to estimate the block length, since the resonances of the benzenedimethanol initiator were clearly detectable (see Figure 2 for a representative spectrum). Polymer 1 has a relative block length composition of the A *hard* (l-Lactide) and B *soft* (HL/CL copolymer) ≈60–270–60, and a *hard* volume fraction of about 35%. Polymer 2 has a similar midblock length, but the *hard* PLLA end blocks length is one-half with respect to polymer 1, resulting in a *hard* volume fraction ≈17%. Polymer 3 has the highest molecular weight with an ABA block composition ≈80–330–80 (*hard* volume fraction = 32%). Finally, polymer 4 has a ≈120–90–120 composition, resulting in a reversed prevailing phase, with a hard volume fraction ≈72%.

**Figure 2:**
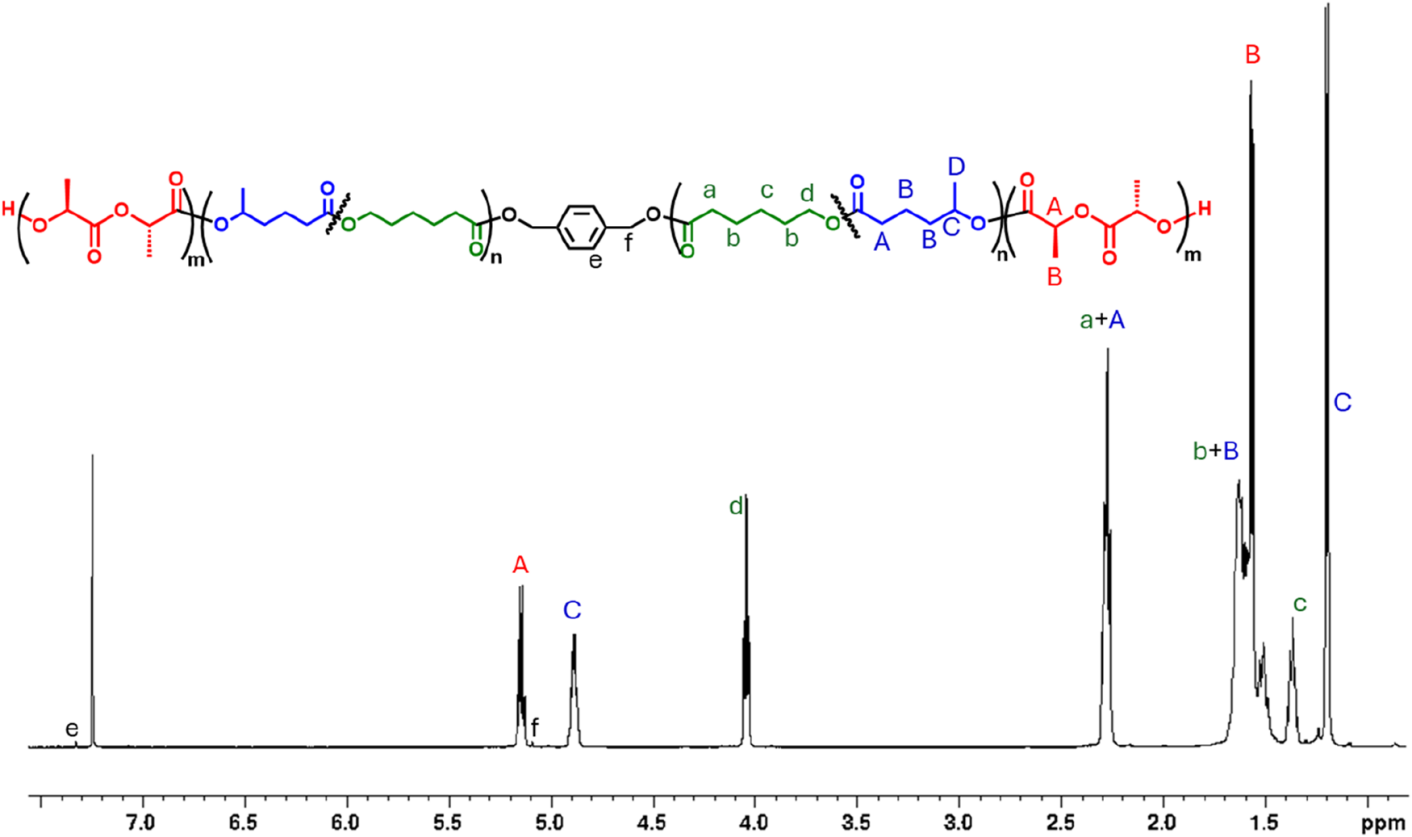
^1^H NMR (CDCl_3_, 600 MHz) spectrum of a poly­(L-LA-*b*-δ-HL-*stat*-ε-CL-*b*-L-LA) triblock polymer (polymer 1 of Table 2).

The ^13^C and 2D ^1^H–^13^C HSQC NMR spectra were also fully assigned (see Figures S6–S7). Furthermore, 2D NMR DOSY experiments confirmed the formation of the triblock copolymers, since the resonances due to the different monomers as well as those due to the initiator exhibited the same diffusion coefficient (see Figure S8).

### Differential Scanning Calorimetry (DSC)

As previously found for the HL/CL copolymers, DSC analysis (see Figure 3) revealed a single glass transition temperature (*T*_g_) in good agreement with the values predicted by the Fox equation based on those of PCL (−60 °C) and HL (−37 °C), thus confirming the random copolymerization of HL and CL. In the triblock copolymers, the *T*_g_ value is not influenced by the presence of PLA, indicating that the hard and the soft blocks are immiscible (see below for further discussion on the matter). This is a fundamental requirement for the formation of thermoplastic elastomers. Crystallization of the PLA segments was observed in the triblock copolymers: for polymer 2, having the lowest fraction of PLA, the melting temperature (*T*_m_) is broad and associated with low enthalpy (6 J/g). As expected, a marked increase in melting enthalpy is detected while the PLA content increases, as longer lactide segments favor crystallization. This behavior directly impacts the mechanical properties of the resulting materials as discuss in the following paragraph.

**Figure 3:**
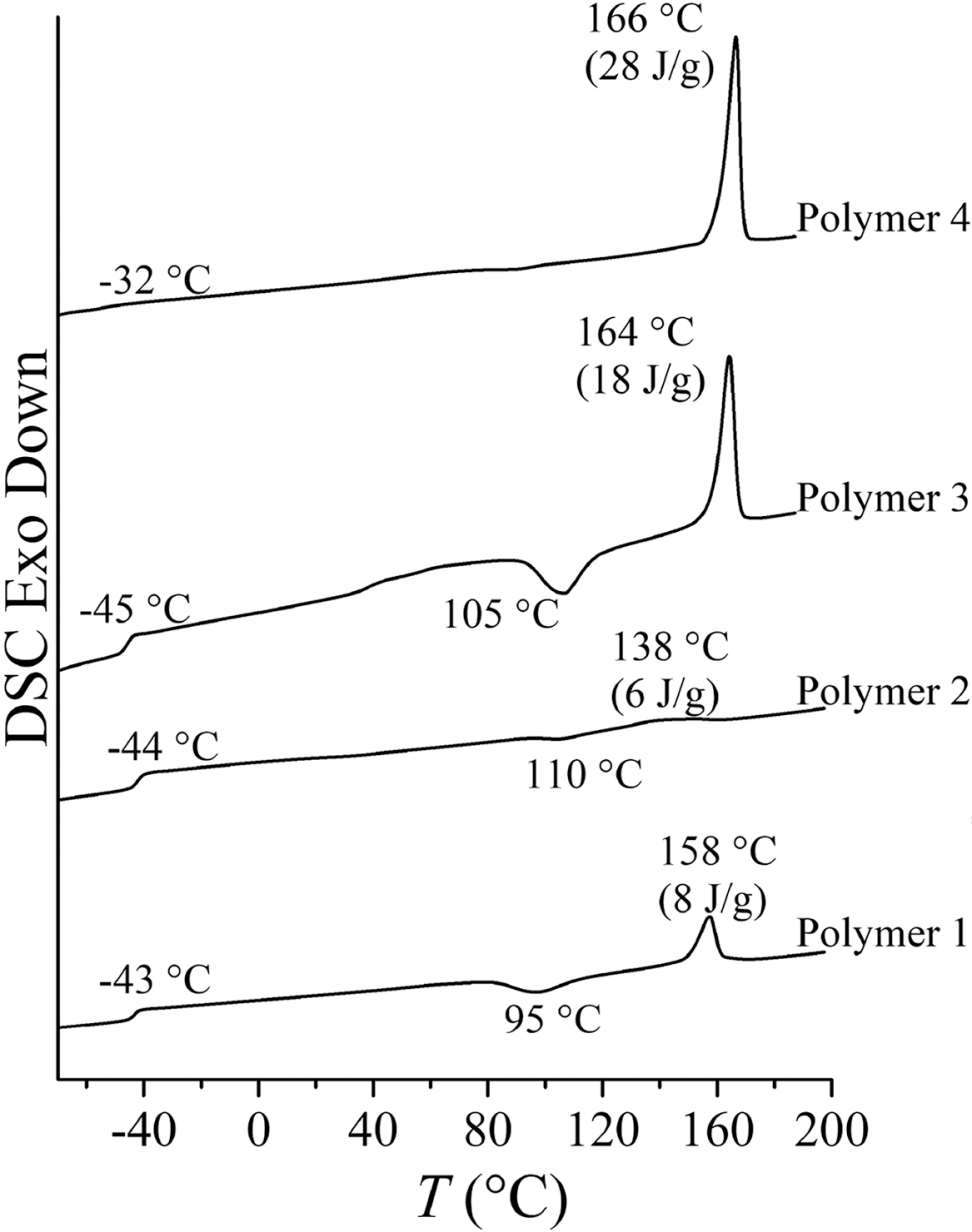
DSC thermograms obtained during the second heating scan of triblock polymers 1–4.

It is worth noting that, in addition to the endothermic melting peak observed in the 138–166 °C range, the DSC second heating scans of polymer 1, polymer 2, and polymer 3 also exhibit an exothermic peak at lower temperatures, attributable to cold crystallization arising from the intrinsically slow crystallization kinetics of PLA. Polymer 4, which has the highest PLA content (72%) and the strongest tendency to crystallize, does not display a cold-crystallization event, indicating that crystallization is essentially complete during the preceding cooling cycle.

By contrast, polymer 1, with a lower PLA content (35%), shows a cold-crystallization peak at approximately 95 °C during the heating scan, suggesting that crystallization upon cooling from the melt is incomplete or largely suppressed. Polymer 3 (hard volume fraction 34%) exhibits a cold-crystallization peak at a slightly higher temperature (∼105 °C), consistent with a further delay in crystallization during reheating and in agreement with its lower PLA content relative to polymer 4 and polymer 1. For polymer 2, which has the lowest PLA weight fraction and overall low crystallinity, both exothermic and endothermic transitions appear as weak and broad features in the DSC heating trace; nevertheless, a broad cold-crystallization peak is still observed at around 110 °C, in line with the general behavior observed across the series.

Overall, these results indicate that the occurrence and temperature of cold crystallization depend not only on the cooling rate but also on the molecular architecture, composition, and PLA weight fraction of the block copolymers, which collectively govern their crystallization behavior.

### Small-Angle X-ray Scattering (SAXS) and Wide-Angle X-ray Diffraction (WAXD) Analysis

Simultaneous SAXS and WAXD profiles were collected during cooling from the melt (200 °C) to room temperature, with a controlled cooling rate of 10 °C/min, using synchrotron radiation to shed light on the morphology and structure of selected PLA-*b*-P­(HL-*stat*-CL)-*b*-PLA triblock samples. SAXS profiles collected in the melt state (Figure 4A), at 200 °C, reveal a clear phase separated morphology for polymer 3 and polymer 4. In particular, polymer 3 showed a well-defined main reflection (*q* ^*^) at 0.185 nm^–1^ followed by higher order reflections at 0.318 and 0,482 nm^–1^ positioned at √3q* and positioned at √7q*, respectively, consistent with a morphology of hexagonally packed PLA cylinders (with a domain spacing *d* of 34 nm) within a δ-hexalactone/ε-caprolactone matrix. This result is consistent with the PLA volume fraction (34 vol %), which lies within the compositional window characteristic of a cylindrical morphology. Polymer 1, with slightly higher PLA volume fraction (35 vol %) and with lower molecular weight presents a SAXS profile almost featureless with a broad and very weak peak q* at ≈0.25 nm^–1^ indicating possibly a weakly phase-separated melt and the absence of long-range order. It is worth noting that SAXS profiles collected during cooling (see Supporting Information) show that the intensity of the scattering peak begins to increase at temperatures below 200 °C, above the PLA crystallization temperature, and continues to grow during PLA block crystallization. This behavior indicates that a phase-separated morphology is stabilized at temperatures below 200 °C and is further driven by PLA crystallization, which increases the effective block incompatibility. However, in this case, no well-defined cylindrical morphology is observed, consistent with the lower molecular weight of this sample compared to polymer 3, which reduces the χ*N* value. Polymer 4 presents the highest content of hard PLA blocks (72 v/v%) and a total molecular weight similar to that of polymer 1. This sample presents a phase-separated melt as demonstrated by the presence of a peak at q* 0.20 nm^–1^ in the SAXS profile of Figure 4A recorded at 200 °C. The absence of additional correlations reflections indicate the absence of long-range order preventing an accurate definition of the morphology, however, a characteristic correlation distance of *d* = 31.4 nm can be estimated from the primary peak position. The absence of long-range order is not uncommon in these systems and can be related to the equilibration kinetics, molar mass and degree of segregation.^28,29^ SAXS profiles collected on samples crystallized during cooling from the phase-separated melt (200 °C) to room temperature (Figure 4B) confirm that the three analyzed samples preserve the phase-separated morphology upon PLA crystallization since polymer 1 still presents a correlation SAXS peak at ≈0.21 nm^–1^ with higher intensity respect to that observed at 200 °C; polymer 3 at room temperature confirm morphological features related to an ordered cylindrical morphology, as clearly evidenced by the presence of correlation peaks at positions q*, √3q*, 2q*, and √7q*, and also for polymer 4 phase separation persists in the crystallized state at room temperature, as evidenced by the retention of the SAXS peak at ≈0.19 nm^–1^. This sample, having the highest amount of PLA fraction, presents an additional weak peak in the SAXS profile indicated with *L* at about 0.33 nm ^–1^ (*d* = 19 nm) which corresponds to the long period of crystalline PLA lamellar stacks. WAXD data collected simultaneously during cooling from the melt confirm the presence of crystalline PLA mainly in α phase as evidenced by the presence of (111), (200), (203), and (015) reflections in the WAXD profiles of Figure 4C. Combined SAXS and WAXS results indicate that PLA crystallization does not destroy the phase-separated morphology present in the melt; instead, crystallization develops within the pre-existing phase-separated domains. WAXD data collected on the isolated midblock copolymer confirm the amorphous behavior (see Supporting Information Figure S2).

**Figure 4:**
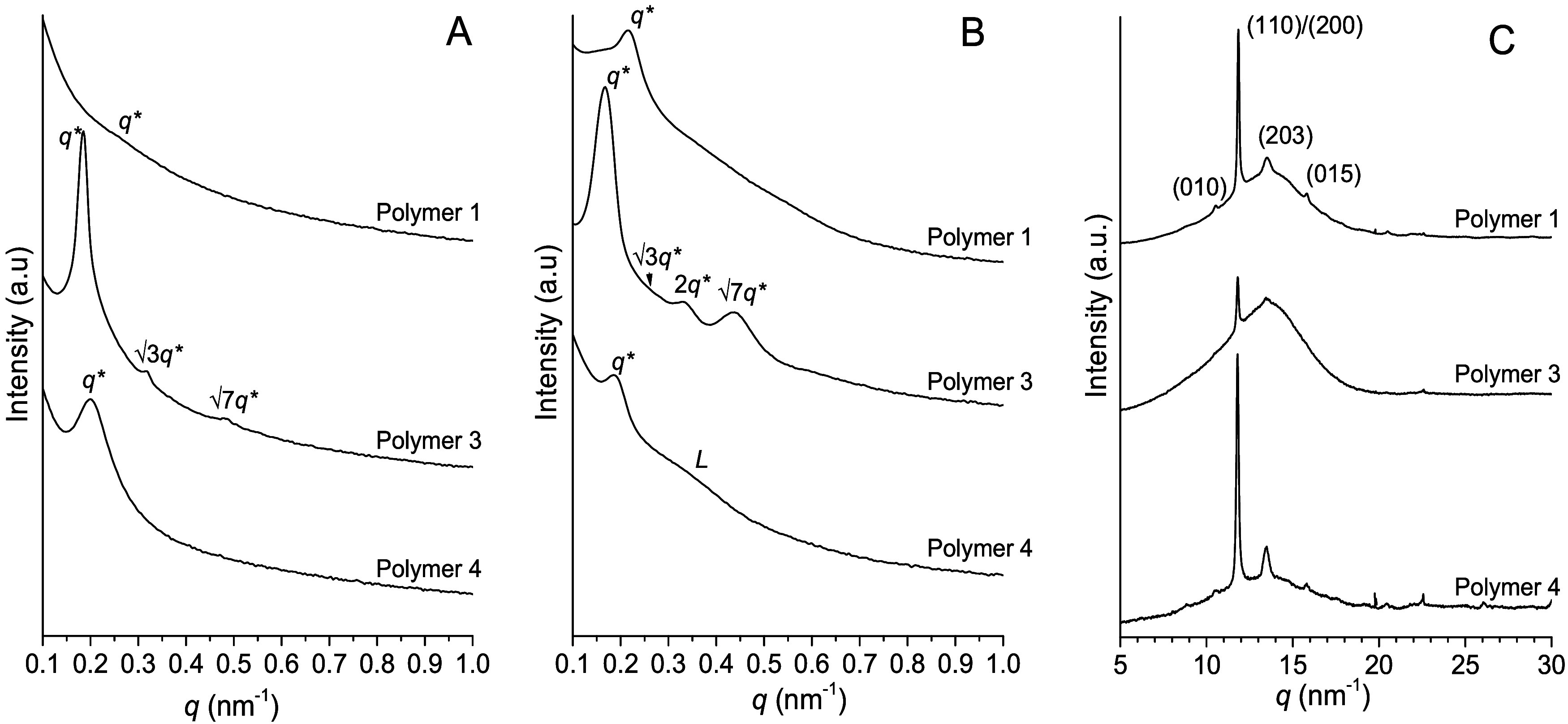
Small angle X-ray scattering (SAXS) profiles collected at 200 °C (melt state) (A), at room temperature on samples crystallized during cooling (10 °C/min) from the melt (B). WAXD data recorded on the melt crystallized samples, collected simultaneously with SAXS profiles reported in B (C).

Similar results were observed in the SAXS profiles recorded at room temperature on films prepared from casting solutions using CH_2_Cl_2_ (Figure 5A). SAXS peaks indicate that a phase-separated morphology is present in all three analyzed samples, revealing a well-defined cylindrical morphology for polymer 3, and a phase-separated morphology without long-range order for polymers 1 and 4. WAXD profiles collected on the same films (Figure 5B) show that polymers 1, 3, and 4 exhibit slight crystallinity when crystallized from solution, compared to the corresponding samples crystallized from the melt (Figure 4C). These data, collected on samples prepared under conditions similar to those used for mechanical testing (crystallized from solution), allow to directly correlate mechanical behavior with morphology and crystallinity.

**Figure 5:**
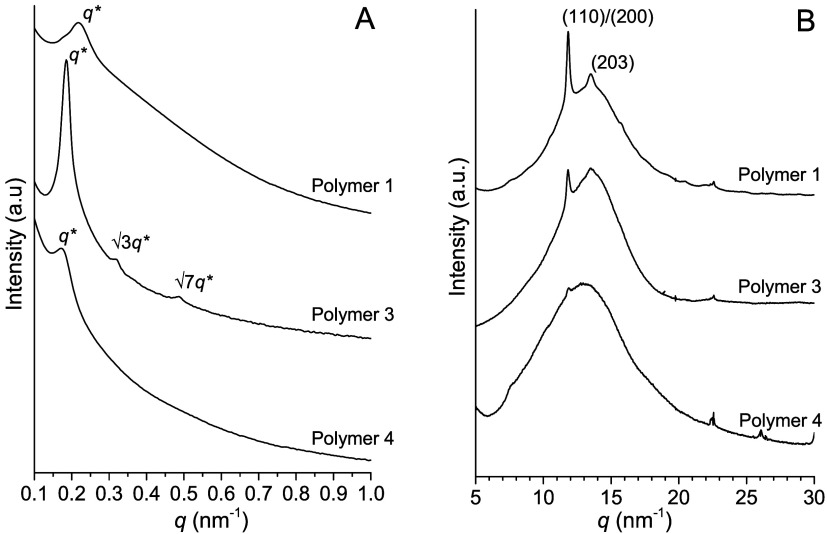
SAXS (A) and WAXD (B) profiles collected at 25 °C on films prepared from casting solutions using CH_2_Cl_2_.

### Mechanical Characterization of Poly­(L-LA-*b*-δ-HL-*stat*-ε-CL-*b*-L-LA)’s

Films of polymers 1–4 prepared by solvent casting were used to perform uniaxial tensile tests (see Figure 6 and Table 2). Polymer 1 (*f*_hard_ = 35%) showed excellent elastomeric behavior, with Young’s modulus > 8 MPa, ultimate tensile strength > 9 MPa, and strain at break > 850%, values comparable to those of some commercial SBS rubbers. In contrast, polymer 2, (*f*_hard_ = 17%), having the same soft midblock, but half hard PLLA end blocks length, behaves like a soft elastomer, showing tensile strength < 1 MPa, strain at break ≈ 170%, Young’s modulus ≈ 4.5 MPa. This strong difference is reasonably due to less efficient physical cross-linking of the short and poorly crystalline (as shown by DSC analysis) PLLA end blocks. As a matter of fact, use of *rac*-LA instead of L-LA in chain extension of soft midblocks used to produce related ABA triblock polymers resulted in amorphous PLA hard end blocks and thus in worse elastomeric properties.^6^ It is worth to note that the relationship between block length and mechanical performance is primarily governed by the volume fraction of the rigid PLA end-blocks, rather than solely the central block length. In A-B-A type block copolymers, the hard A-blocks (PLA) undergo microphase separation to form discrete, rigid domains. These domains serve two critical functions within the soft B-block matrix: they act as physical cross-links, transforming the fluid-like polymer into an elastic solid with an effect which is similar to reinforcing filler particles, significantly increasing the material’s initial stiffness. Thus, the PLA end-blocks (hard domains) dictate the material’s strength and Young’s modulus, analogous to the polystyrene domains in classic Styrene–Butadiene-Styrene (SBS) block copolymers. Conversely, the central B-blocks form the soft, continuous matrix that primarily contributes to the material’s elasticity and toughness. Our data analysis, particularly the direct comparison between polymer 1 and polymer 2, is in perfect agreement with this mechanism, demonstrating that materials with a higher PLA volume fraction exhibit superior modulus and strength, irrespective of the absolute B-block chain length (Table 3).

**Figure 6:**
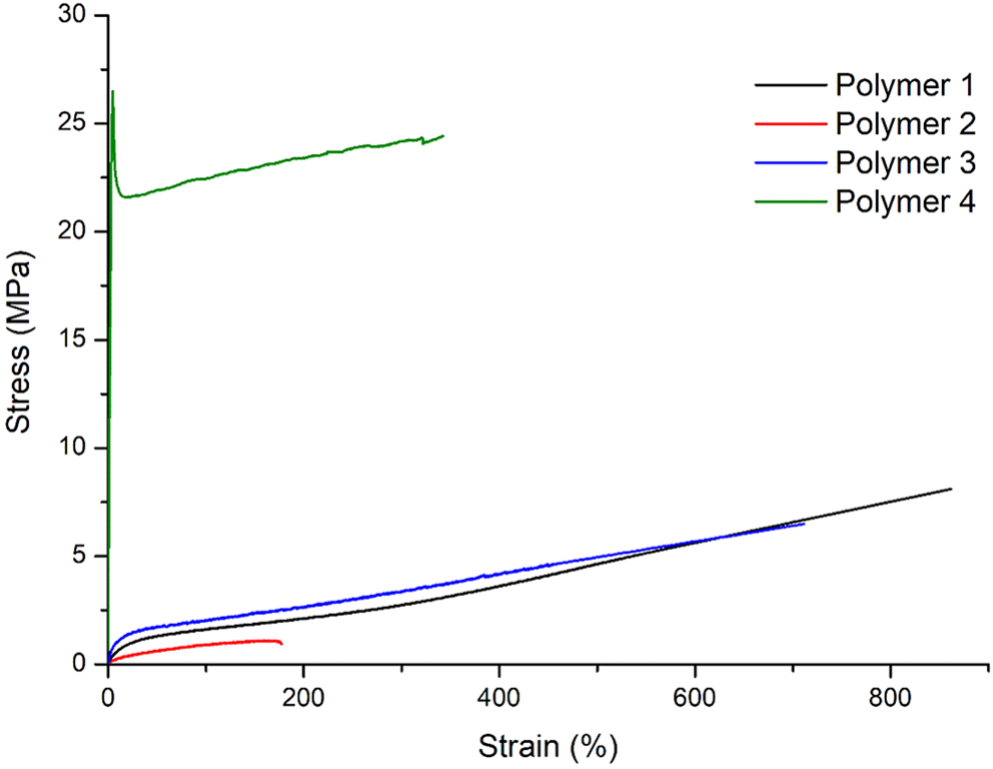
Uniaxial tensile tests for triblock polymers 1–4.

**Table 3:** Effect of the PLA end block length on the the mechanical properties of polymers 1 and 2

polymer	PLA end block length	midblock length	PLA volume fraction	observed modulus
polymer 1	longer	fixed	higher	higher
polymer 2	shorter	fixed	lower	lower

This comparison highlights that an increase in the volume fraction of the rigid PLA end-blocks (polymer 1) leads to a higher density of physical cross-links and a more cohesive hard-domain network, resulting in a significantly increased Young’s Modulus. This effect dominates any minor changes related to the central block length within the studied range.

As far as the ultimate properties are concerned, the same structure–property principles that relate PLA end-block volume fraction to Young’s modulus applies. In particular, the Ultimate Tensile Strength (UTS) is directly governed by the volume fraction of the hard PLA end-blocks, following the same structure—property principles established for Young’s modulus. In A-B-A block copolymers, the UTS reflects the maximum load-bearing capacity and integrity of the microphase-separated A (PLA) domains. These rigid domains function as reinforcing anchors that must be fully ruptured or pulled through the soft matrix for bulk material failure to occur. Thus, increasing the overall volume fraction of the rigid PLA end blocks (as in polymer 1 vs polymer 2) increases the density of the physically cross-linked network created by microphase-separated domains. Moreover, a denser, more continuous hard-domain network more effectively transfers mechanical stress from the soft midblock to the reinforcing PLA domains. In other words, since UTS represents the maximum stress the material can withstand before macroscopic failure, a more robust and interconnected PLA hard-domain network requires substantially greater force to initiate chain pullout, domain rupture, or crack propagation, resulting in a higher UTS.

Polymer 3, having higher MW and *f*_hard_ like polymer 1, also showed excellent elastomeric behavior, with a significantly higher Young’s modulus (24 MPa) and a slightly lower strain at break and ultimate tensile strength compared to polymer 1 (see Table 2). The polymer was subjected to repeated tensile cycles at a strain of 100%. The choice of this strain level was based on a combination of established protocols, current literature practice, and the necessity to obtain application-relevant data while remaining within the defined scope of our study. However, the chosen strain of 100% is high enough to effectively capture the Mullins effect (stress softening), which is crucial for characterizing the stability and energy dissipation properties of thermoplastic elastomers, also providing a clear measurement of energy dissipation (hysteresis loop area) and recovery performance. As a result of tensile cycles, polymer 3 presented an initial hysteresis of approximately 20% deformation, followed, for the subsequent cycles, by a notable elastic recovery of approximately 95%. This recovery, as the number of cycles increases, tends to increase, reaching values close to 98%, with progressive stabilization of the material highlighted by the reduction of the hysteresis area (see Figure S12) The elastomeric behavior is consistent with the phase-separated morphology, which consists of hard PLA domains acting as physical cross-links within the amorphous matrix.

The mechanical behavior of polymer 4, having a *f*_hard_ = 72%, high strain at break (∼300%), yield strength ∼ 28 MPa, ultimate tensile strength ∼ 25 MPa, and Young’s modulus ∼ 0.9 GPa, reflects a toughened, ductile material (Figure S13). This behavior is closely linked to its thermal transitions and morphology, particularly the high fraction of hard domains, its low glass transition temperature (*T*_g_ = −32 °C) and melting transition with a *T*_m_ of 166 °C and melting heat of 28 J/g that results the highest. In this sample, the morphology is characterized by a phase-separated microstructure in which rubbery domains, with a correlation distance of approximately 30 nm (see SAXS in Figure 5A), are dispersed within the hard, slightly crystalline PLA matrix (see WAXD in Figure 5B). These rubbery domains act as a toughening phase, enhancing the material’s ductility and flexibility and thereby influencing its deformation mechanism.

Analysis of the DSC thermograms could suggest an apparent lack of correlation between the low measured melting enthalpy (Δ*H*_m_) and the relatively high Young’s modulus (*E*). However, as noted earlier, the mechanical response is governed less by the degree of crystallinity than by the microphase-separated morphology of the material, much like in classic SBS block copolymers, where the hard polystyrene blocks are amorphous. In the case of polymer 4, the low Δ*H*_m_ simply reflects the limited overall crystallinity of the PLA end blocks, indicating that the crystalline domains are small and imperfect. The comparatively high Young’s modulus (*E*), on the other hand, arises from the physical cross-links established by the hard PLA domains. Regardless of whether these domains are crystalline, amorphous, or partially ordered, they segregate from the soft midblock and act as load-bearing junctions that efficiently resist deformation at small strains, thereby dictating the initial stiffness. This behavior closely parallels that of TPEs such as SBS, where the hard polystyrene domains form discrete, rigid aggregates dispersed in a soft polybutadiene matrix. At low strains, deformation is controlled by the physically cross-linked network created by these hard domains, leading to a high modulus even when the crystalline order is limited (i.e., low Δ*H*_m_). As strain increases, the soft phase elongates until the network begins to yield or the hard domains orient or separate, determining the large-strain properties. In our system, the PLA end blocks play an analogous role, forming a rigid physical network that dominates the small-strain mechanical response, while the DSC data (Δ*H*_m_) merely reflects the modest degree of crystallographic order within those domains.

### Chemical Recycling to the Monomers

Chemical recycling to monomer (CRM) has recently gained attention as a promising strategy for achieving a circular plastics economy.^30^ This approach enables the conversion of postconsumer plastic waste back into its original monomer form, allowing for repolymerization and effectively closing the material loop while reducing reliance on virgin feedstocks. However, the feasibility of CRM is strongly influenced by polymerization thermodynamics,^30,31^ making it more or less challenging depending on the polymer type. On this basis, significant research has been recently directed toward aliphatic polyesters with more favorable thermodynamic parameters,^15^ including δ-lactone (co)­polymers.^16,32^ However, CRM of triblock polymers closely related to ours, e.g., consisting of PLLA hard end blocks and (co)­polymers of δ-substituted-δ-lactones,^16,33^ was not successfully achieved by thermal depolymerization in the presence of Sn­(II) octanoate, owing to the “poor depolymerization selectivity of PLLA”.^16^ In fact, until recently CRM was considered not feasible for PLA,^30^ and different chemical recycling strategies have been extensively investigated. However, more recently efficient CRM of PLLA by simple thermal depolymerization in the presence of a suitable Sn­(II) or Zn­(II) catalyst has been reported.^34,35^ As well documented in the literature,^36–39^ performing the reaction in the presence of a high-boiling alcohol can markedly accelerate depolymerization. Polyols, in particular, are known to enhance depolymerization^35,39^ kinetics by promoting transesterification reactions that reduce polymer molecular weight and diminish melt-viscosity effects. Under these conditions, the larger number of hydroxyl termini facilitates the formation of tin or zinc alkoxides, the catalytically active species. A second mechanism proposed in the literature involves coordination of hydroxyl groups to the tin center, which increases the nucleophilicity of chain-end S*n*-alkoxides. This interaction lowers the activation barrier for backbiting reactions, thereby promoting monomer formation.

We thus evaluated the degradation of a poly­(L-LA-*b*-δ-HL-*stat*-ε-CL-*b*-L-LA) sample (polymer 2 of Table 2) by isothermal TGA experiments at 180 °C under a nitrogen flow of 40.0 mL min^–1^ using Sn­(Oct)_2_ or Zn­(Oct)_2_ either in presence or in absence of a high boiling alcohol, glycerol ethoxylate (Figures S14–S16). Comparative analysis of the TGA data revealed that Sn­(Oct)_2_ in combination with GEO, with a ratio [OH]/[catalyst] of 10:1, exhibited the highest catalytic activity, yielding a kinetic constant of 70 h^–1^ (see Figure 7).

**Figure 7:**
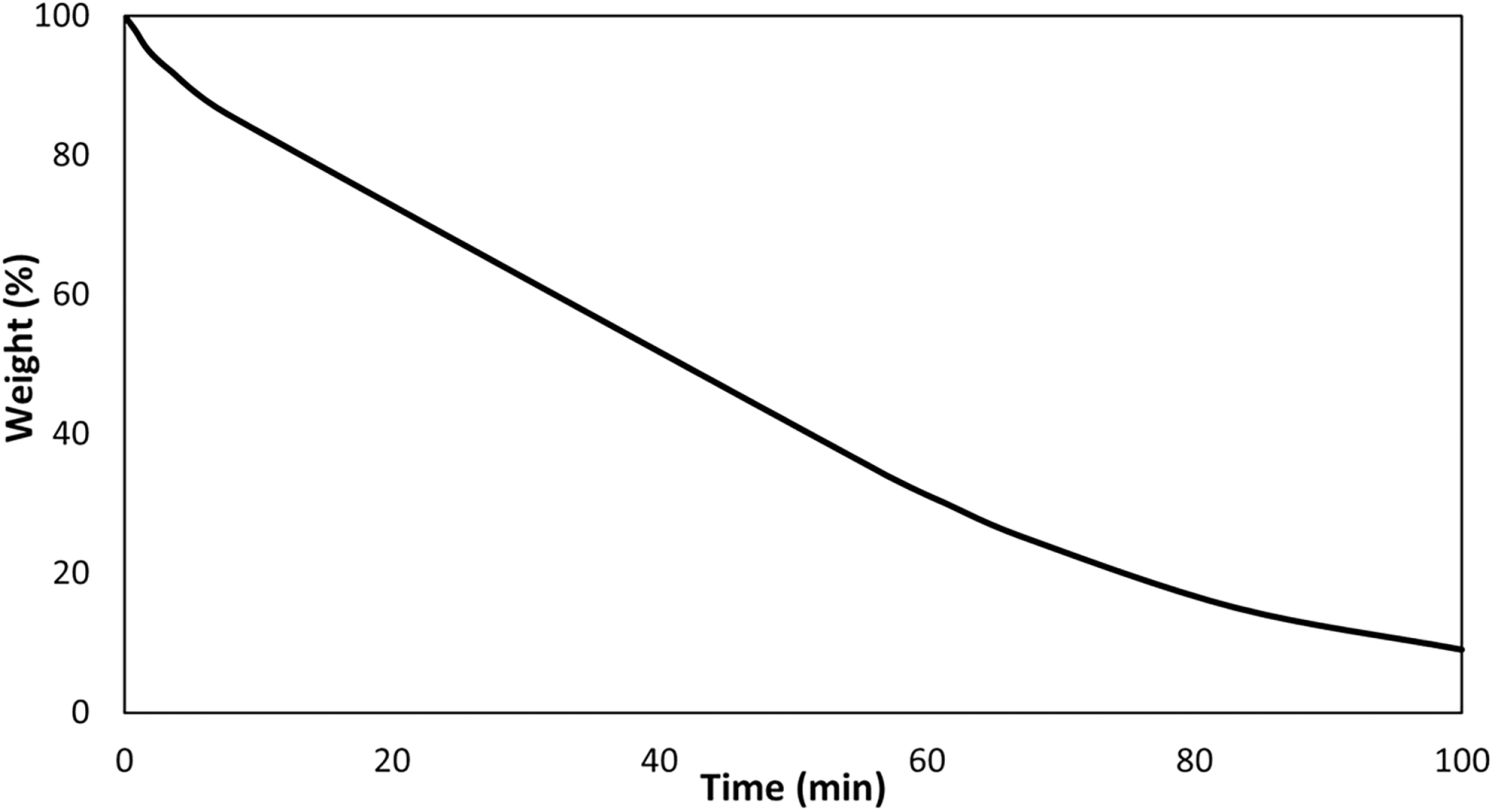
Evolution of mass loss of a triblock polymer sample (polymer 2) over the time from TGA experiments.

After suitable degradation conditions were identified, bulk depolymerization tests were carried out, heating 0.100 g of the triblock polymer 2 at 180 °C for 2 h in a distillation apparatus with magnetic stirring under vacuum (<1 Torr) in the presence of Sn­(Oct)_2_ (2 mol % with respect to ester groups) and GEO ([OH]/[Sn] = 10:1). The reaction products were collected in a flask immersed in liquid nitrogen and were analyzed by ^1^H NMR, showing high recovery of the monomers with high purity: >99% of lactide, 96% of δ-hexalactone and 94% of ε-caprolactone (see Figure 8). These preliminary results demonstrate that the synthesized poly­(L-LA-*b*-δ-HL-*stat-*ε-CL-*b*-L-LA) can be fully depolymerized back to its constituent monomers, aligning with emerging principles in sustainable polymer science, where materials are engineered not only for high performance but also for controlled chemical recycling at end-of-life.

**Figure 8:**
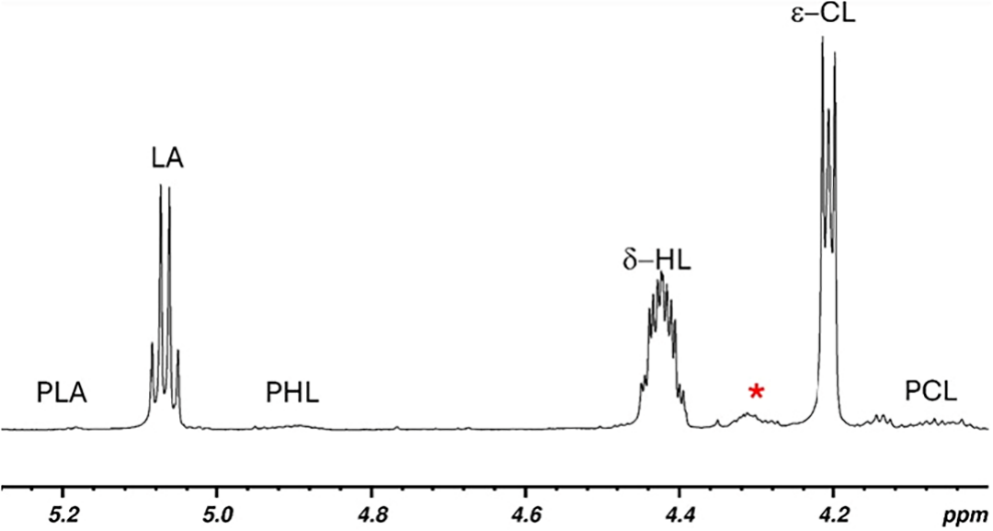
^1^H NMR (CDCl_3_, 298 K, 400 MHz) spectrum of the mixture resulting from catalytic thermal depolymerization of a poly­(L-LA)-*b*-(δ-HL-*stat*-ε-CL)-β-(L-LA) triblock copolymer. **red asterisk** transesterification adduct GEO-polymer.

## Conclusions

In this work we have demonstrated that the combination of iron-based catalysis and a one-pot strategy enables the efficient synthesis of ABA triblock copolymers composed of semicrystalline poly­(l-lactide) hard segments and amorphous δ-hexalactone/ε-caprolactone statistical copolymers as the soft midblock. The use of δ-substituted lactones, which are naturally occurring and readily available on an industrial scale, proved particularly advantageous, as it allowed the preparation of low-*T*_g_, noncrystalline midblocks, a fundamental requirement for well-performing thermoplastic elastomers. The synthesized triblock copolymers exhibited highly tunable mechanical properties, governed by the block composition and the relative phase fraction. Systematic variation of the molecular architecture enabled access to a wide spectrum of material behaviors, ranging from soft, silicone-like elastomers with low modulus and high extensibility, to styrene–butadiene-styrene (SBS)-like rubbers combining high tensile strength with outstanding elastic recovery. At higher hard-block content, the copolymers approached toughened polylactide (PLA) analogues,^40^ displaying enhanced rigidity and impact resistance. This broad mechanical tunability underscores the versatility of the system and highlights its potential for applications spanning from sustainable elastomers to high-performance engineering thermoplastics. A key outcome of this study is the demonstration of the chemical recyclability of the triblock copolymers. Catalytic depolymerization under mild conditions enabled near-quantitative recovery of the constituent monomers, confirming the feasibility of closing the polymer life cycle. This efficient monomer recovery validates the potential of the system for circular material design, offering a tangible route toward minimizing environmental impact and advancing the circular polymer economy.

Overall, this study demonstrates that δ-substituted lactones offer a versatile and promising platform for the development of renewable, recyclable, and performing thermoplastic elastomers. Future research will aim to expand the monomer library, deepen the understanding of composition-property relationships, and address challenges related to thermal processing. Additionally, efforts will focus on optimizing chemical recycling conditions to advance the transition from fossil-based elastomers to greener, circular alternatives with reduced environmental impact.

## Supplementary Material





## Notes

^1^H, ^13^C NMR spectra and 2D-DOSY NMR for triblock characterization, GPC profiles used to evaluate triblock formation, supplementary SAXS patterns of triblock copolymers during cooling from melt, supplementary tensile stress–strain curves, evolution of mass loss of triblock copolymers obtained from TGA experiments, a supplementary photograph of films obtained by solution casting (PDF)
Video illustrating the different mechanical behavior of a thermoplastic elastomer (polymer 1) and a toughened PLA (polymer 4) (MP4)
