# Supplementary material for: Sustainable Thermoplastic Elastomers from Commercial Cyclic Esters: One-Pot Synthesis of Poly(l‑lactide)‑b‑(δ-hexalactone-stat-ε-caprolactone)‑b‑(l‑lactide) and Its Chemical Recycling to the Monomers
Source: ACS Polym Au. 2026 Jan 8;6(1):468–79. doi: 10.1021/acspolymersau.5c00172 (PMC12903435; doi:10.1021/acspolymersau.5c00172)
Supplement: Supplementary file 1 [file lg5c00172_si_001.pdf]

Supporting Information

Sustainable Thermoplastic Elastomers from Commercial Cyclic Esters: One-pot Synthesis of Poly(L-lactide)-b-(δ-hexalactone-stat-ε-caprolactone)-b-(L-lactide) and its Chemical Recycling to the Monomers

Giuseppe Gravina,[1] Joseph N. A. Tagoe,[2] Rocco Di Girolamo,[3] Maria Gentile,[1,4] Luciano Di Maio[2],

Claudio Pellecchia[1]*

[1] Dipartimento di Chimica e Biologia "A. Zambelli", Università degli Studi di Salerno, via Giovanni Paolo II 132, Fisciano, SA 84084, Italy.

[2] Dipartimento di Ingegneria Industriale, Università degli Studi di Salerno, via Giovanni Paolo II 132, Fisciano, SA 84084, Italy.

[3] Dipartimento di Scienze Chimiche, Università degli Studi di Napoli Federico II, Via Cintia 21, Napoli 80126, Italy.

[4] Dipartimento di Ingegneria Industriale, Università di Padova, via Gradenigo, 6/a, Padova, PD 35131, Italy

Email: cpellecchia@unisa.it

Table of Contents

Figure S1: Carbonyl region of the ^{13}C NMR spectrum of the HL/CL copolymer produced in run 3 of Table 1.

Figure S2: WAXD pattern of the δ-hexalactone/ε-caprolactone midblock copolymer used to prepare polymers 1 and 2 of **Table 4**.

Figure S3: GPC profiles for polymers 1 and 2 compared to the isolated midblock before chain extension with L-lactide, confirming chain growth in the second step.

Figure S4: GPC profiles for polymer 3 compared to the isolated midblock before chain extension with L-lactide, confirming chain growth in the second step.

Figure S5: GPC profiles for polymer 4 compared to the isolated midblock before chain extension with L-lactide, confirming chain growth in the second step.

Figure S6: ^{13}C NMR spectrum ($CDCl_3$, 600 MHz) of poly(LA-*b*-δ-HL-*stat*-ε-CL-*b*-LA) (Polymer 3, Table 4).

Figure S7: HSQC NMR spectrum ($CDCl_3$, 600 MHz) of poly(LA-*b*-δ-HL-*stat*-ε-CL-*b*-LA) (Polymer 3, Table 4).

Figure S8: DOSY NMR spectrum ($CDCl_3$, 600 MHz) of poly(LA-*b*-δ-HL-*stat*-ε-CL-*b*-LA) (Polymer 3, Table 4).

Figure S9: SAXS patterns of polymer 1 (Table 4) acquired during cooling from the melt (200°C) to room temperature at a cooling rate of 10 °C/min (1 profile per °C).

Figure S10: SAXS patterns of polymer 3 (Table 4) acquired during cooling from the melt (200°C) to room temperature at a cooling rate of 10 °C/min (1 profile per °C).

Figure S11: SAXS patterns of polymer 3 (Table 4) acquired during cooling from the melt (200°C) to room temperature at a cooling rate of 10 °C/min (1 profile per °C).

Figure S12: Tensile profile for stress-strain cycles of polymer 3 (Table 4).

Figure S13: Magnified view of the stress-strain profile at low strain for polymer 4 (Table 4).

Figure S14: Evolution of poly(LA-*b*-δ-HL-*stat*-ε-CL-*b*-LA)mass loss over the time from TGA experiments in presence of 2 mol% of $Sn(Oct)_2$ relative to ester linkages (Run 1 of Table S1).

Figure S15: Evolution of poly(LA-*b*-δ-HL-*stat*-ε-CL-*b*-LA) mass loss over the time from TGA experiments using 2 mol% of $Zn(Oct)_2$ relative to ester linkages (Run 2 of Table S1).

Figure S16: Evolution of poly(LA-*b*-δ-HL-*stat*-ε-CL-*b*-LA) mass loss over the time from TGA experiments using 2 mol% of $Zn(Oct)_2$ relative to ester linkages and GEO with a ratio [OH]:[catalyst] of 10:1 (Run 4 of Table S1).

Table S1: Depolymerization of poly(LA-*b*-δ-HL-*stat*-ε-CL-*b*-LA) from TGA experiments.

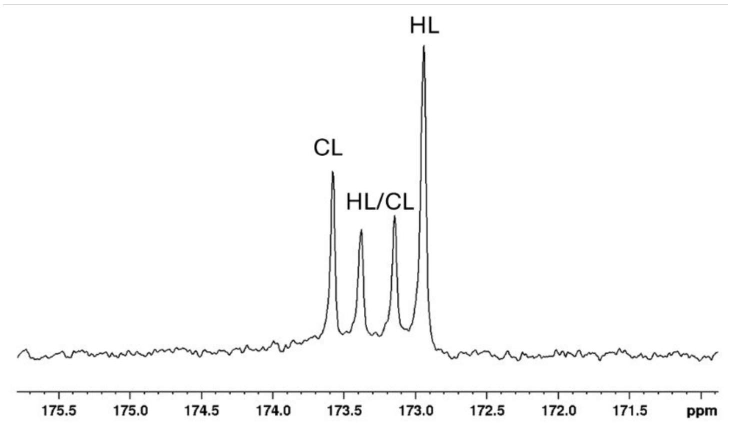

Figure S1: Carbonyl region of the ^{13}C NMR spectrum of the HL/CL copolymer produced in run 3 of **Table 1**.

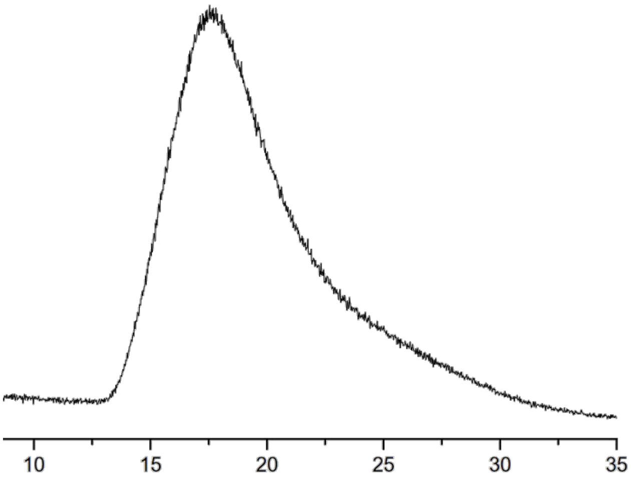

Figure S2: WAXD pattern of the δ-hexalactone/ε-caprolactone midblock copolymer used to prepare polymers 1 and 2 of **Table 4.**

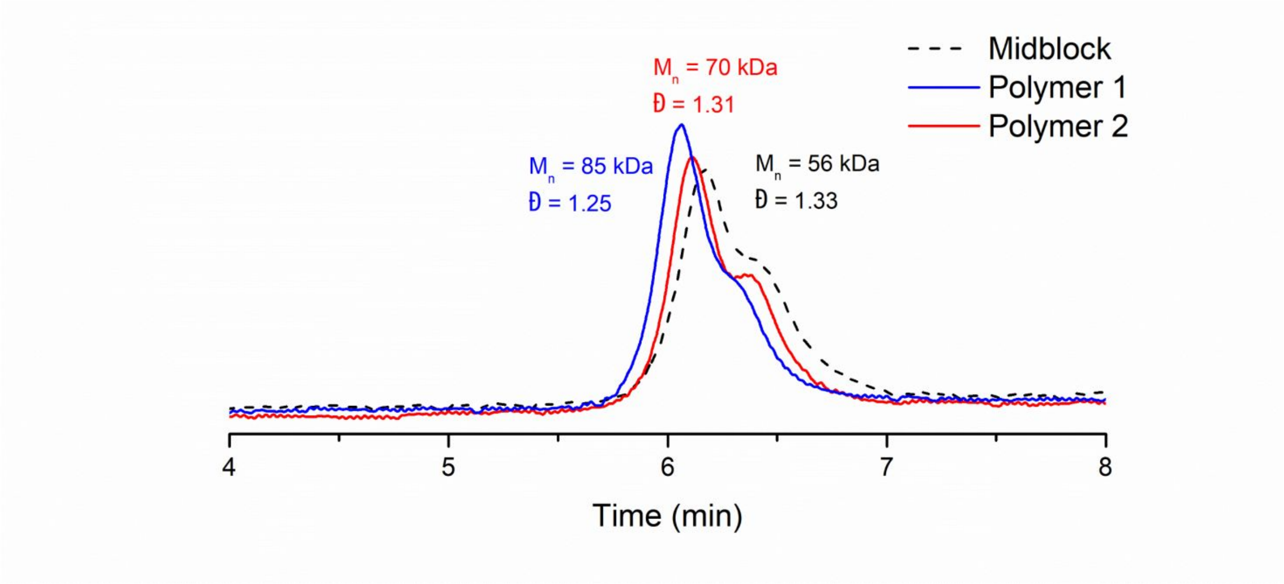

Figure S3: GPC profiles for polymers 1 and 2 compared to the isolated midblock before chain extension with L-lactide, confirming chain growth in the second step.

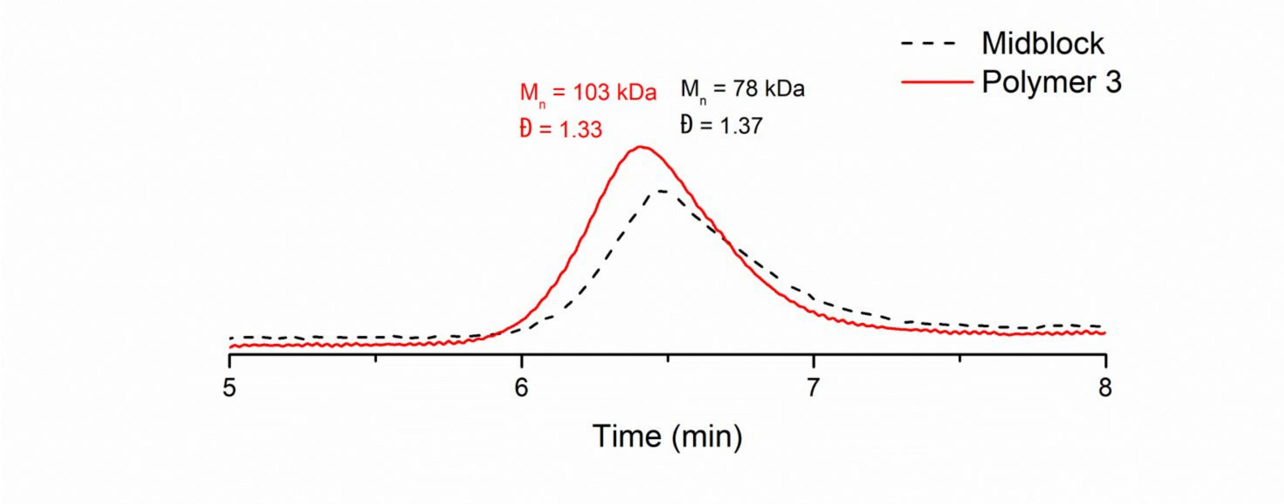

Figure S4: GPC profiles for polymer 3 compared to the isolated midblock before chain extension with L-lactide, confirming chain growth in the second step.

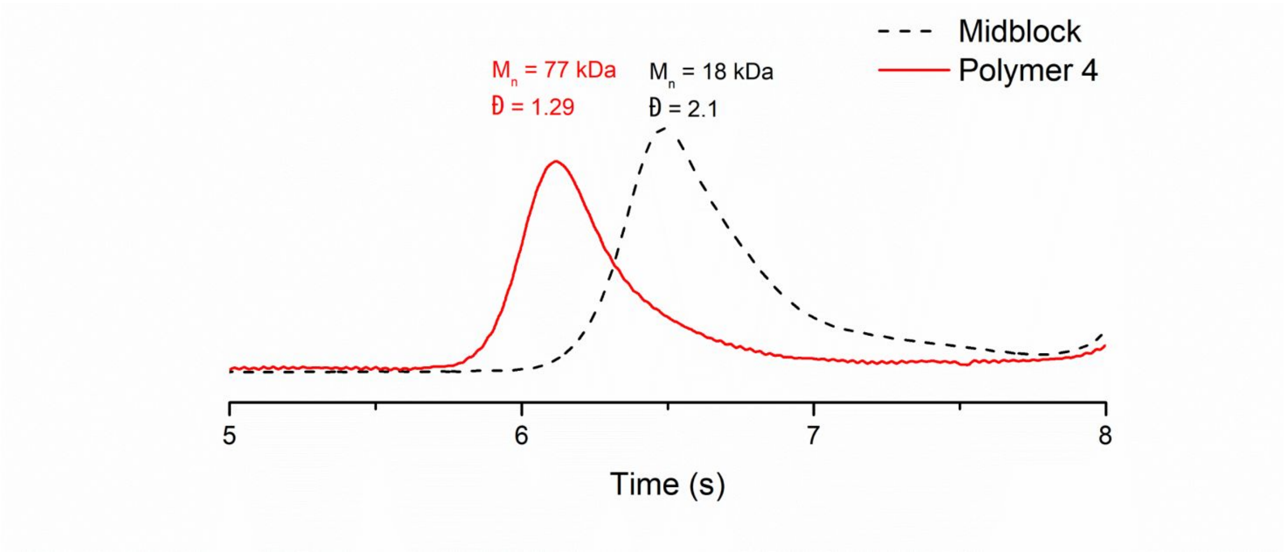

Figure S5: GPC profiles for polymer 4 compared to the isolated midblock before chain extension with L-lactide, confirming chain growth in the second step.

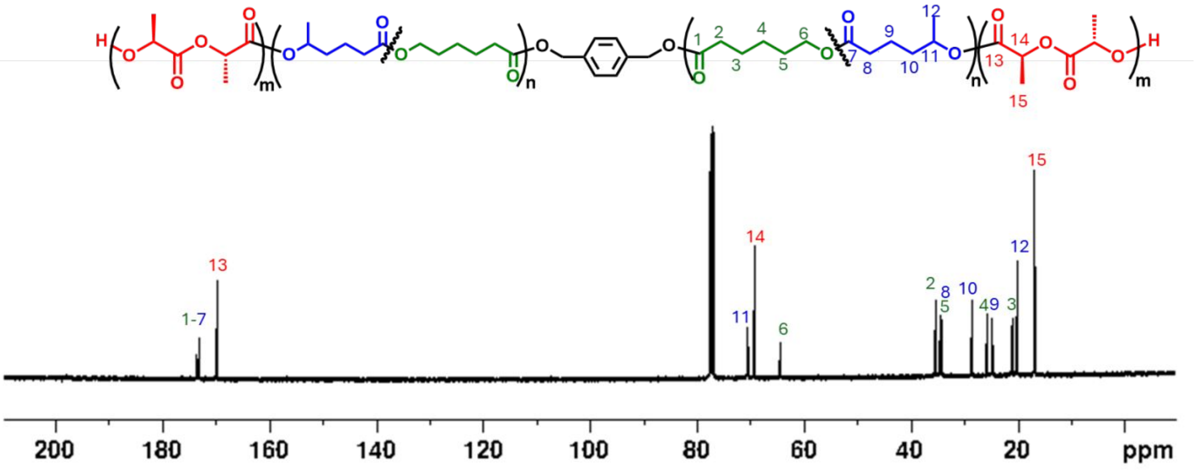

Figure S6: ^{13}C NMR ($CDCl_3$, 600 MHz) of poly(LA-*b*-δ-HL-*stat*-ε-CL-*b*-LA) (Polymer 3, Table 4)

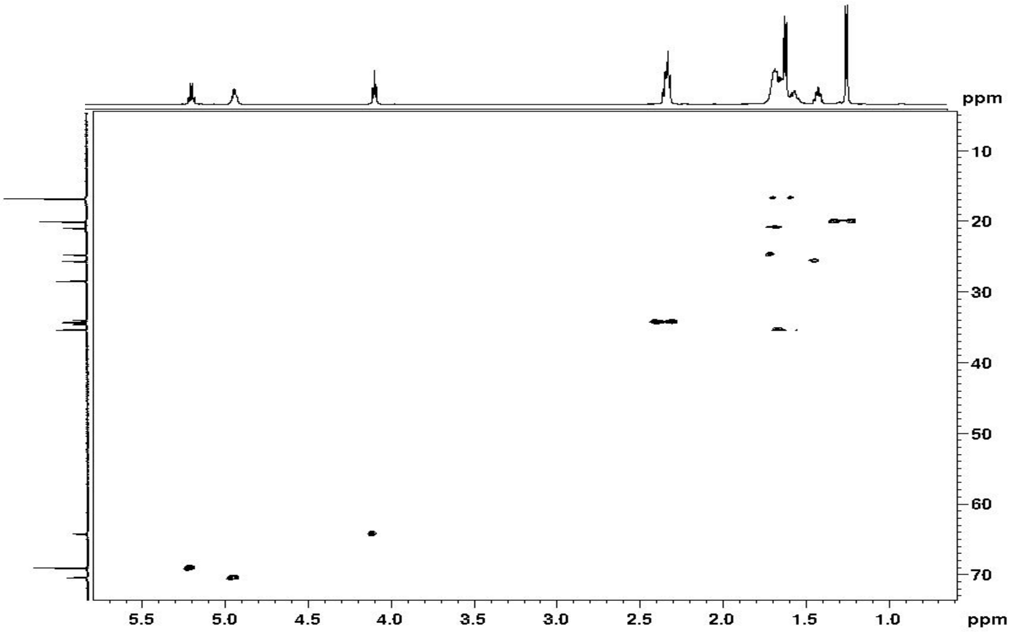

Figure S7: HSQC ($CDCl_3$, 600 MHz) of poly(LA-*b*-δ-HL-*stat*-ε-CL-*b* -LA) (Polymer 3, Table 4)

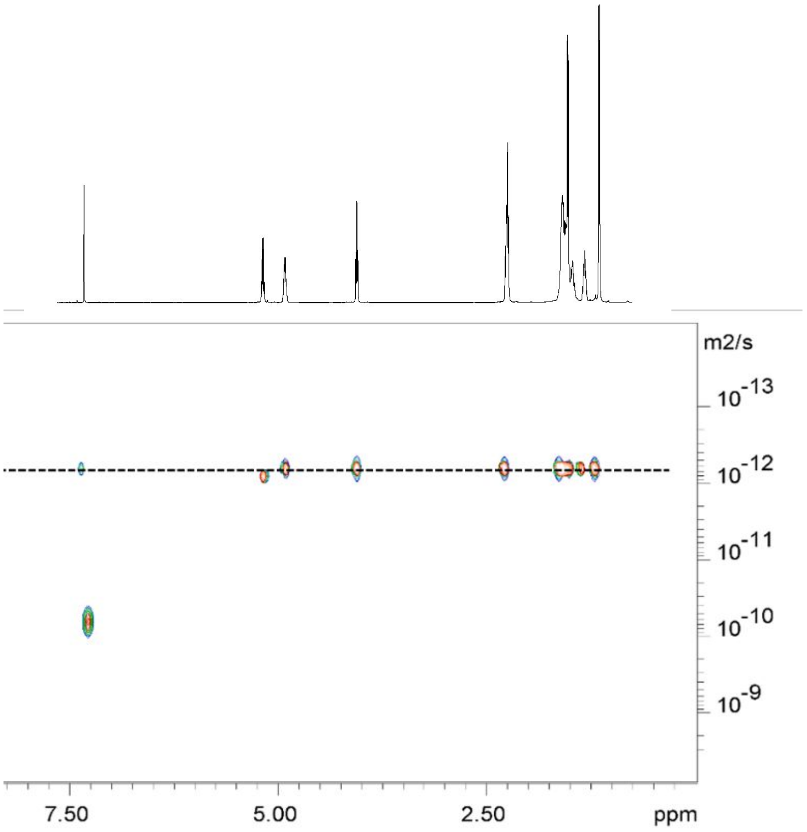

Figure S8: DOSY NMR spectrum ($CDCl_3$, 600 MHz) of poly(LA-*b*-δ-HL-*stat*-ε-CL-b-LA) (Polymer 3, **Table 4**)

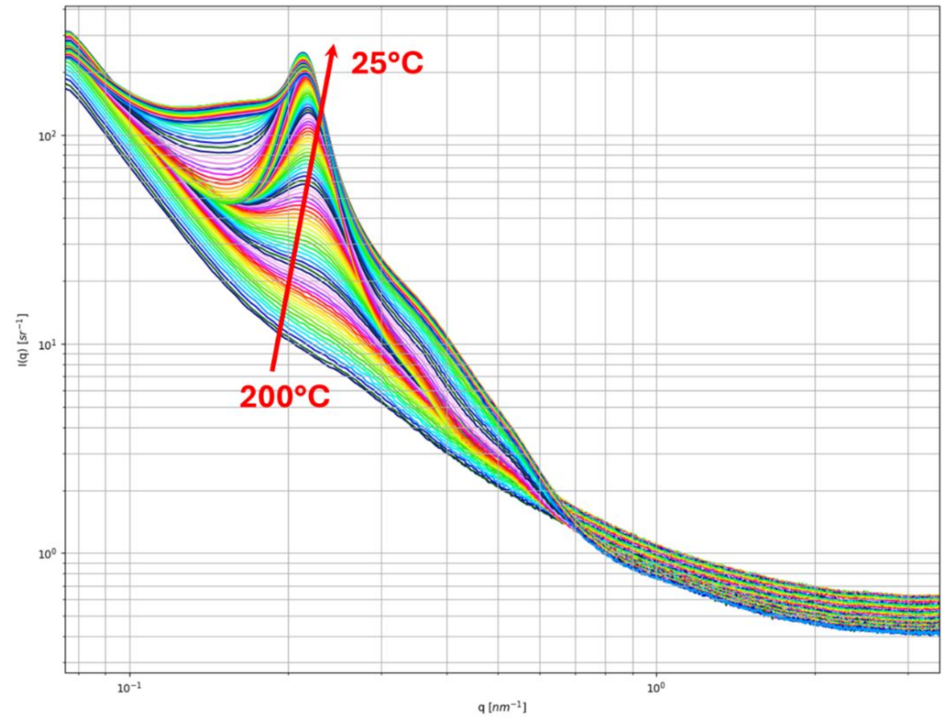

Figure S9: SAXS patterns of Polymer 1 acquired during cooling from the melt (200°C) to room temperature at a cooling rate of 10 °C/min (1 profile per °C).

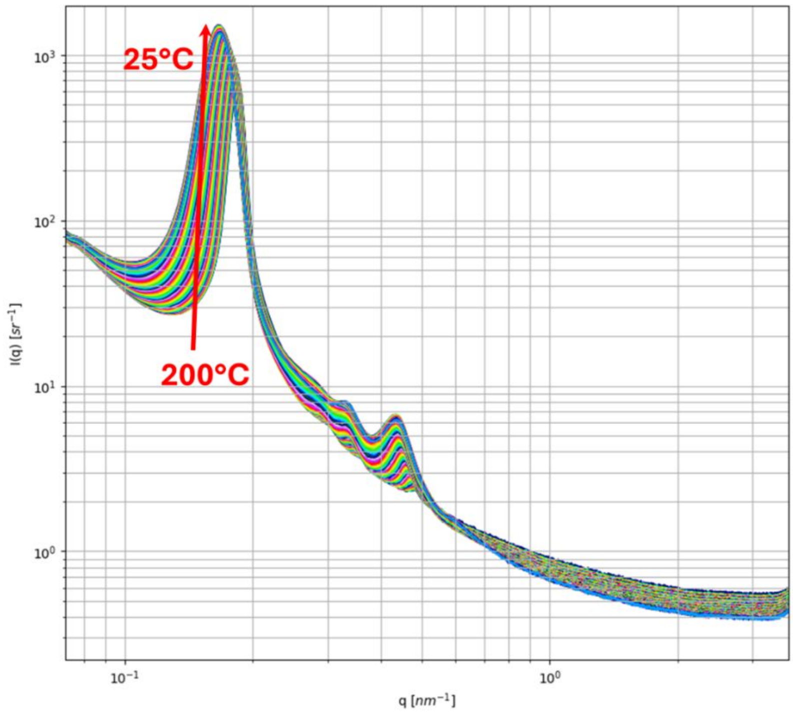

Figure S10: SAXS patterns of Polymer 3 acquired during cooling from the melt (200°C) to room temperature at a cooling rate of 10 °C/min (1 profile per °C).

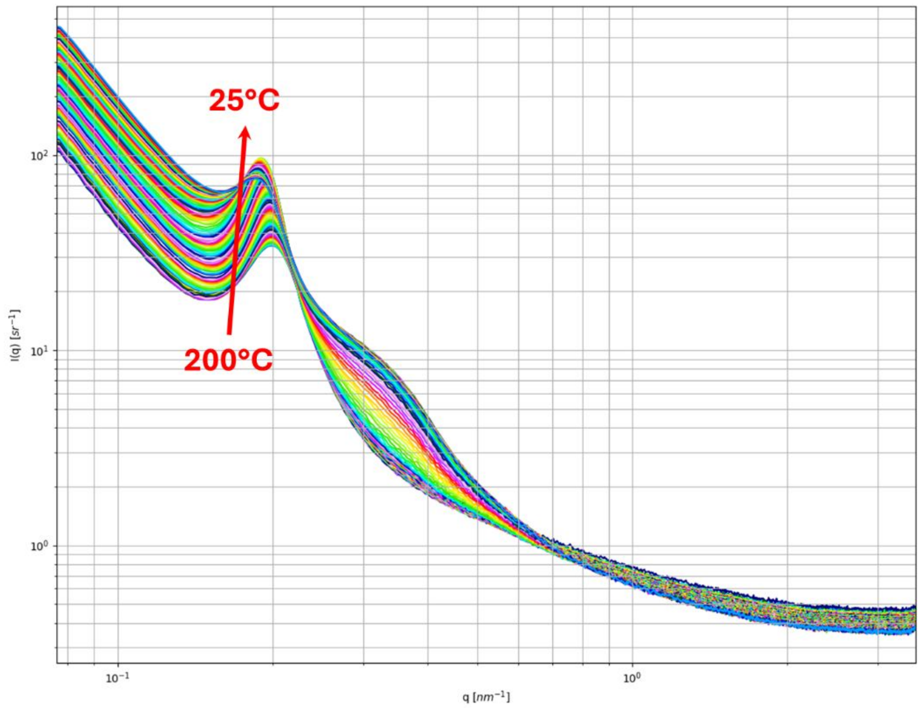

Figure S11: SAXS patterns of Polymer 4 acquired during cooling from the melt (200°C) to room temperature at a cooling rate of 10 °C/min (1 profile per °C).

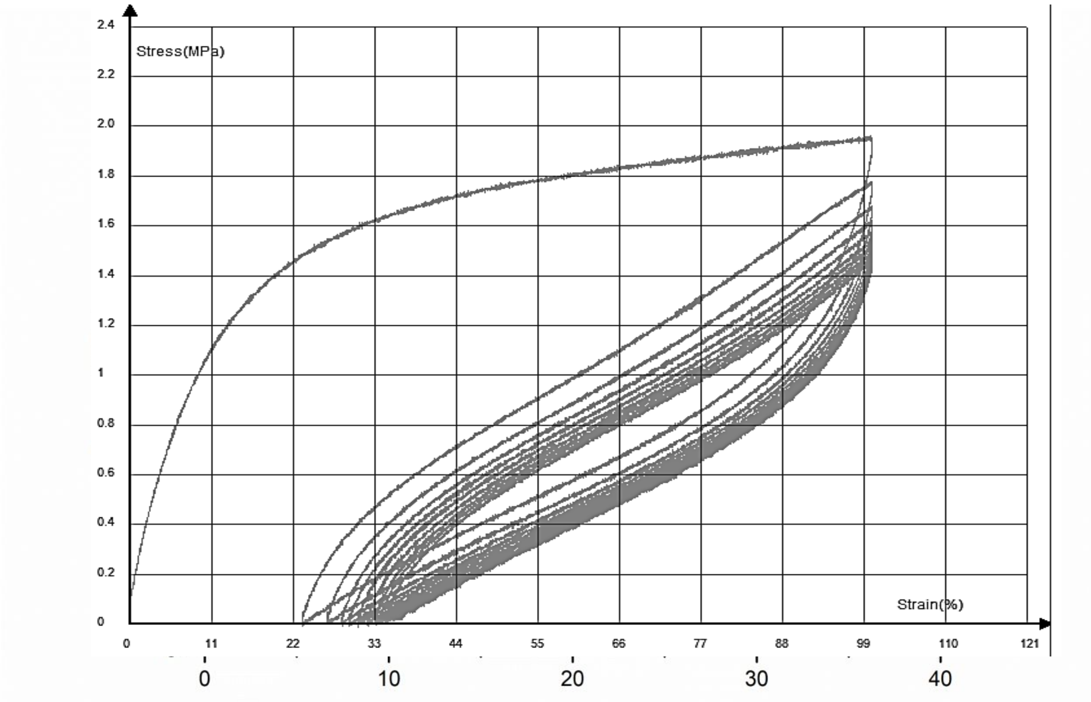

Figure S12: Tensile profile for stress-strain cycles of triblock polymer 3 of **Table 4**

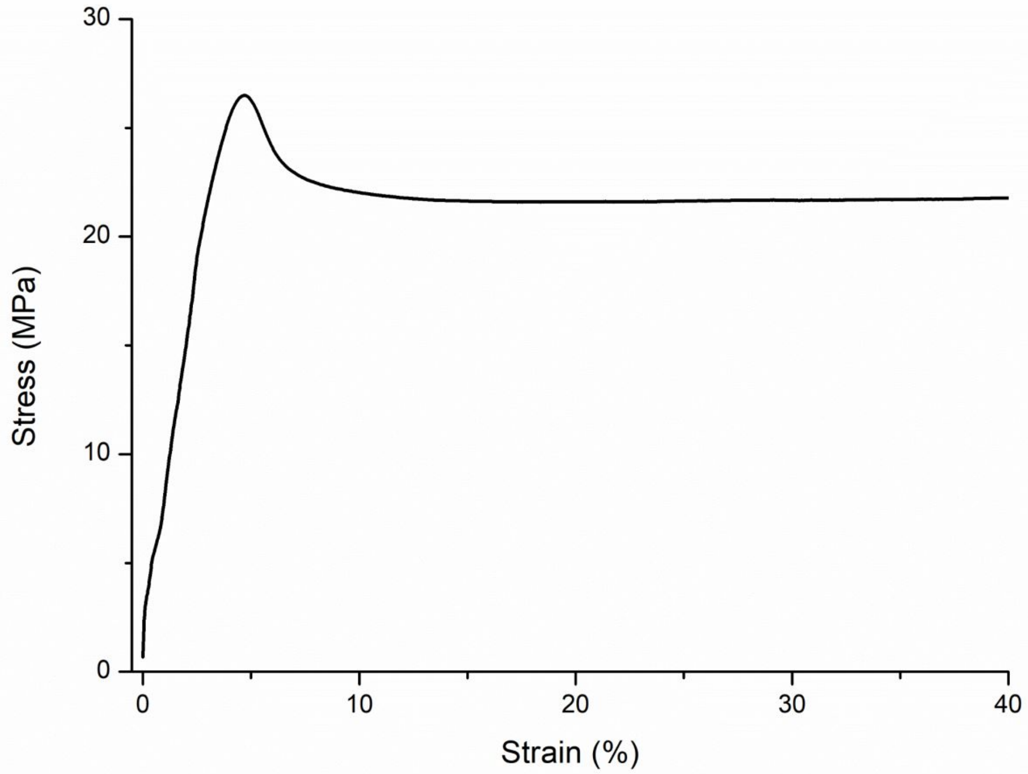

Figure S13: Magnified view of the stress-strain profile at low strain for polymer 4.

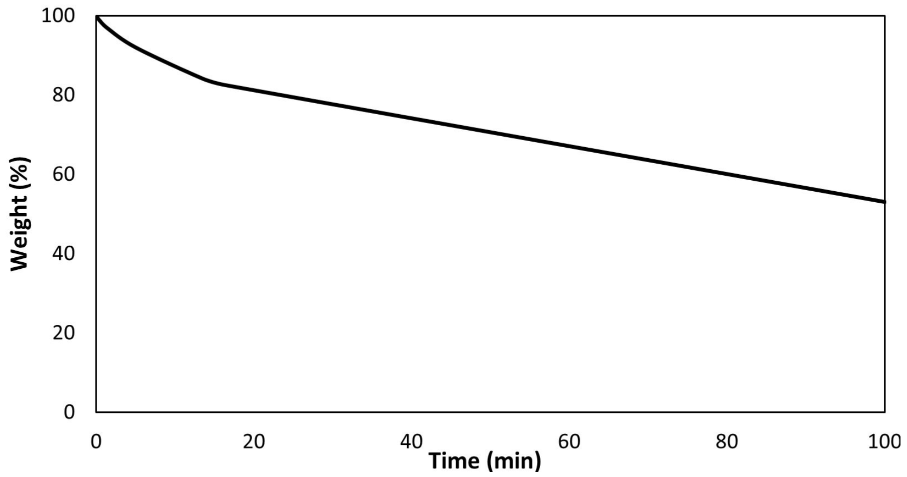

Figure S14: Evolution of PLA-b-P(HL-co-CL)-b-PLA mass loss over the time from TGA experiments in presence of 2 mol% of $Sn(Oct)_2$ relative to ester linkages (Run 1 **Table S1**).

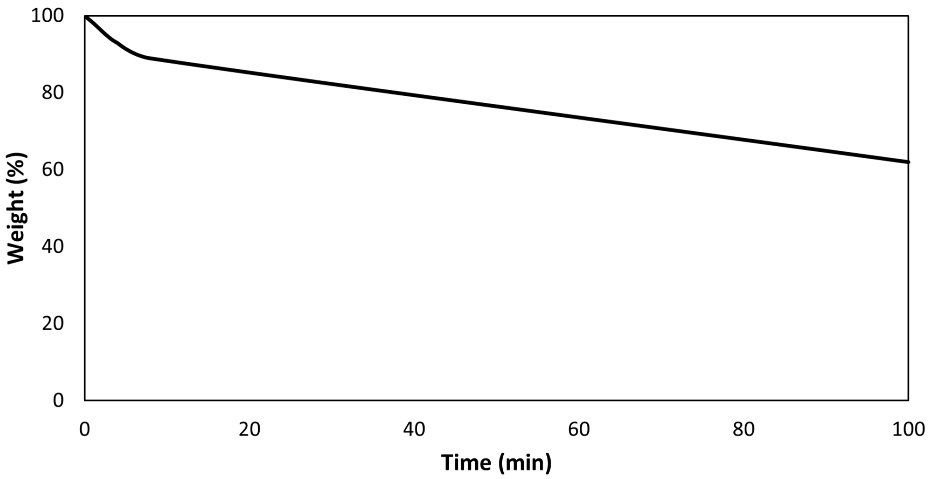

Figure S15: Evolution of PLA-b-P(HL-co-CL)-b-PLA mass loss over the time from TGA experiments using 2 mol% of $Zn(Oct)_2$ relative to ester linkages (Run 2 **Table S1**).

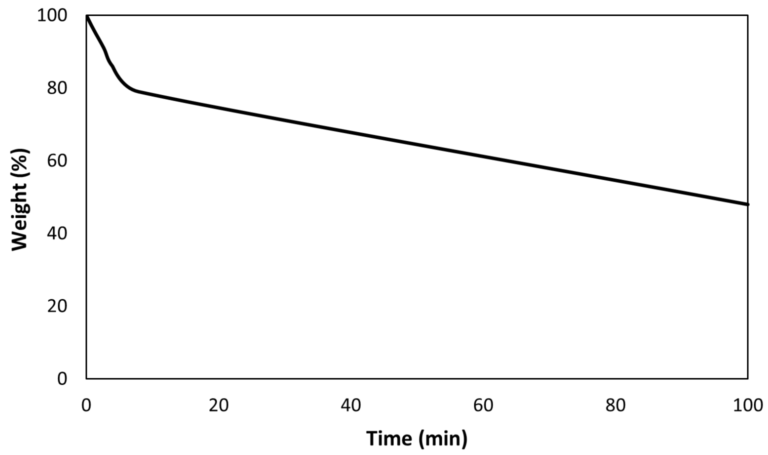

Figure S16: Evolution of PLA-b-P(HL-co-CL)-b-PLA mass loss over the time from TGA experiments using 2 mol% of $Zn(Oct)_2$ relative to ester linkages and GEO with a ratio [OH]:[catalyst] of 10:1 (Run 4 **Table S1**).

Table S2: Depolymerization of PLA-b-P(HL-co-CL)-b-PLA from TGA experiments

Run[a]	Catalyst	Alcohol[b]	k[h^{-1}][c]	R^2
1	**$Sn(Oct)_2$**	/	26 h^{-1}	0,997
2	**$Zn(Oct)_2$**	/	20 h^{-1}	0,996
3	**$Sn(Oct)_2$**	GEO	70 h^{-1}	0,998
4	**$Zn(Oct)_2$**	GEO	35 h^{-1}	0,998

[a]0.003 g of polymer **2** of Table 2; 2 mol% (relative to ester linkages) of catalyst; T=180°C, 100minutes of isothermal; [b]($[GEO]_{OH}$:[cat] = 10:1), [c] the rate constant is the gradient of the linear fits to the plots of % polymer **2** mass loss over the time estimated by TGA.

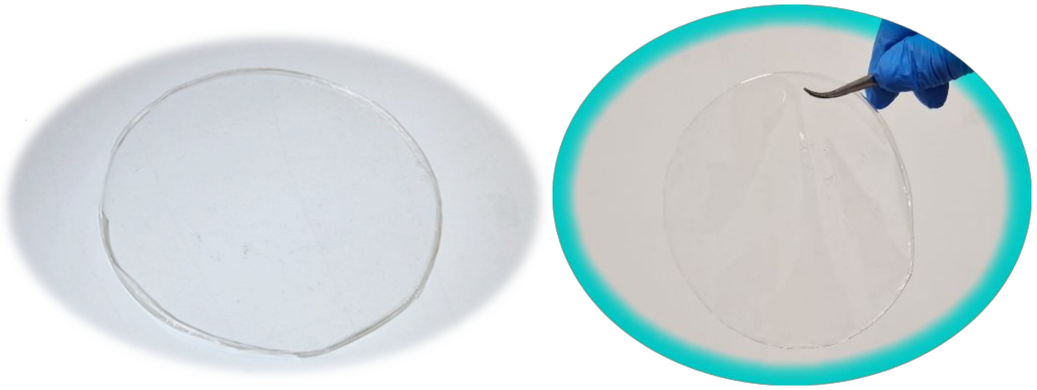

Figure S17: Photographs of the obtained triblock copolymer films. An additional supplementary video is added.
